# Network Pharmacology-Based Validation of Caveolin-1 as a Key Mediator of Ai Du Qing Inhibition of Drug Resistance in Breast Cancer

**DOI:** 10.3389/fphar.2018.01106

**Published:** 2018-10-02

**Authors:** Neng Wang, Bowen Yang, Xiaotong Zhang, Shengqi Wang, Yifeng Zheng, Xiong Li, Shan Liu, Hao Pan, Yingwei Li, Zhujuan Huang, Fengxue Zhang, Zhiyu Wang

**Affiliations:** ^1^The Research Center of Basic Integrative Medicine, Guangdong Provincial Academy of Chinese Medical Sciences, Guangzhou University of Chinese Medicine, Guangzhou, China; ^2^Integrative Research Laboratory of Breast Cancer, Discipline of Integrated Chinese and Western Medicine, The Second Affiliated Hospital of Guangzhou University of Chinese Medicine, Guangzhou, China; ^3^Tropical Medicine Institute, Guangzhou University of Chinese Medicine, Guangzhou, China

**Keywords:** breast cancer chemosensitivity, network pharmacology, bioinformatics analysis, Ai Du Qing, caveolin-1

## Abstract

Chinese formulas have been paid increasing attention in cancer multidisciplinary therapy due to their multi-targets and multi-substances property. Here, we aim to investigate the anti-breast cancer and chemosensitizing function of Ai Du Qing (ADQ) formula made up of *Hedyotis diffusa*, *Curcuma zedoaria (Christm.) Rosc.*, *Astragalus membranaceus (Fisch.) Bunge*, and *Glycyrrhiza uralensis Fisch*. Our findings revealed that ADQ significantly inhibited cell proliferation in both parental and chemo-resistant breast cancer cells, but with little cytotoxcity effects on the normal cells. Besides, ADQ was found to facilitate the G2/M arresting and apoptosis induction effects of paclitaxel. Network pharmacology and bioinformatics analysis further demonstrated that ADQ yielded 132 candidate compounds and 297 potential targets, and shared 22 putative targets associating with breast cancer chemoresponse. Enrichment analysis and experimental validation demonstrated that ADQ might improve breast cancer chemosensitivity *via* inhibiting caveolin-1, which further triggered expression changes of cell cycle-related proteins p21/cyclinB1 and apoptosis-associated proteins PARP1, BAX and Bcl-2. Besides, ADQ enhanced *in vivo* paclitaxel chemosensitivity on breast cancer. Our study not only uncovers the novel function and mechanisms of ADQ in chemosensitizing breast cancer at least partly *via* targeting caveolin-1, but also sheds novel light in utilizing network pharmacology in Chinese Medicine research.

## Introduction

Breast cancer is the most common female malignancy and one of the leading causes of cancer-related deaths, with 252,710 new cases of invasive breast cancer and 40,610 breast cancer-related deaths diagnosed in women in the United States in 2017 (DeSantis et al., 2017). Chemotherapy is one of the main therapeutic approaches for treating this disease, but it remains non-selectively toxic to normal tissues (Li et al., 2005). Paclitaxel (taxol) is the first-line treatment for metastatic breast cancer. The activity of paclitaxel is primarily due to its inhibitory effects on microtubule assembly, which leads to arrest of the mitotic phase of the cell cycle and subsequent apoptosis. Nevertheless, paclitaxel application is still limited due to its systemic toxicity and acquired resistance (Janczar et al., 2017; Stage et al., 2018). Thus, there is an urgent need for novel and safe chemosensitizing strategies. Currently, increasing attention has been paid to the synergistic effects of natural phytochemicals in enhancing the chemoresponse and relieving cytotoxic effects (Wang et al., 2014).

Traditional Chinese Medicine (TCM) has attracted worldwide attention from clinicians and researchers due to its multi-target and multi-substance characteristics and good safety profile (Gupta et al., 2013). In contrast to Western medicine, TCM prescriptions are composed of several herbs and are called formulas. Originating from Yellow Emperor’s Classic theory (Chinese name Huang Di Nei Jing), the golden principle of formula composition should be based on Jun-Chen-Zuo-Shi (Lu, 1985; Fan et al., 2006). The ADQ formula was created by Prof. Zhiyu Wang based on the Jun-Chen-Zuo-Shi principle and long-term clinical experience. The ADQ formula is mainly composed of four herbs: Jun (Emperor) herb *Hedyotis diffusa* (Chinese name Bai Hua She Cao, BHSSC), Chen (Minister) herb *Curcuma zedoaria (Christm.) Rosc.* (Chinese name E Zhu, EZ), Zuo (adjuvant) herb *Astragalus membranaceus (Fisch.) Bunge* (Chinese name Huang Qi, HQ), and Shi (courier) herb *Glycyrrhiza uralensis Fisch* (Chinese name Gan Cao, GC). Each herb in the formula can inhibit cancer growth *via* cell cycle arrest, apoptosis induction, and immune regulation (Fu et al., 2014a; Gao et al., 2014; Wang et al., 2014, 2015b; Feng et al., 2017). In addition, each herb is a drug that is frequently prescribed to cancer patients according to statistical analyses of the medication rules of national TCM masters (Song et al., 2015). However, the multi-target and multi-substance properties of this formula have made it very challenging to explore its underlying mechanisms.

In the past decade, multi-omic technologies including genomics, transcriptomic, proteomics, metabolomics, and serum pharmacokinetics have been developed for the high-throughput screening and identification of targets involved in TCM formulas (Li et al., 2014). However, these traditional approaches are expensive and require multidisciplinary collaboration and complex analytical procedures (Xu et al., 2017). With the development of bioinformatics, systems biology is emerging as a more holistic approach for integrating compound–target interactions from a molecular to system level. One of the most significant applications of systems biology is to use network pharmacology to understand the complex mechanism of actions of TCM formulas. Following ingredient collection and screening, pharmacokinetic evaluation (absorption, distribution, and metabolism), target prediction, and network analysis (Liu et al., 2013), it is becoming faster and easier to present an entire drug-target interaction network and determine the involved core molecule and pathways. In addition, by intersecting with a disease target database, it is more efficient to elucidate how formulas intervene with critical targets that facilitate disease occurrence and progression (Ru et al., 2014).

The current study was designed to determine the preclinical efficacy of ADQ against breast cancer *in vitro* and *in vivo*. To this end, we investigated the anti-cancer and chemosensitizing functions of ADQ extracts in this disease. The results showed that ADQ effectively and safely enhanced breast cancer chemosensitivity *in vitro* and *in vivo*. To determine the mechanism of action, we constructed a “drug–target–disease” network among ADQ components (drug), ADQ targets (target), and genes in breast cancer chemoresistance (disease). The results of the network analysis and biological experimental findings suggested that ADQ mainly targets CAV1 to induce chemosensitizing effects. The results of this study not only provide scientific evidence to support the application of ADQ formula in the treatment of breast cancer but also highlight the novel role of network pharmacology in the modernization of TCM.

## Materials and Methods

### Preparation and Quality Control of ADQ

For ADQ preparation, BHSSC, EZ, HQ, and GC were mixed at a 1:1:1:1 ratio and then subjected to a grinding machine. The mixture was extracted with 95% alcohol by reflux extraction for 1 h and repeated three times. Then the supernatants were concentrated by rotary evaporation and evaporated to dryness in a water bath to obtain raw ethanol extract powder. The production ratio was calculated as 7.2–9.6%. For quality control analysis, the Agilent 1260 System (Agilent, Palo Alto, CA, United States) with DAD was applied for HPLC analysis. The Agilent C_18_ Column (5 μm, 250 mm × 4.6 mm) with the SecurityGuard Cartridge System (Phenomenex, Sacramento, CA, United States) was applied for HPLC analysis. The mobile phases consisted of acetonitrile (A) and 0.05% (v/v) phosphoric acid (B) using a gradient program of 15% A in 0–23 min, 15–38% A in 23–40 min, 38% A in 40–50 min, 38–61% A in 50–60 min, and 61% in 60–75 min. The flow rate was 1.0 mL/min and the column temperature was set to 30°C The DAD detector was set at 216, 236, 260, 276, and 308 nm. P-coumaric acid, calycosin-7-glucoside, liquiritin, glycyrrhizic acid, and curcumol were prepared and diluted with methanol for the preparation of standard solutions. A volume of 10 μL of these solutions was analyzed with HPLC, and the calibration curves were finally established. For the preparation of sample solutions, 0.1 g ADQ was dissolved in 20 mL methanol. After sonicating for 60 min, the sample solution was filtrated through a 0.2 μm membrane filter for HPLC analysis.

### Cell Culture

The human breast cancer cell lines MDA-MB-231 and MCF-7 were purchased from the American Type Culture Collection (Manassas, VA, United States). MDA-MB-231/T and MCF-7/T cells were derived from parental cells by gradually increasing paclitaxel (taxol) treatments for 6 months in the laboratory. MDA-MB-231, MCF-7, MDA-MB-231/T, and MCF-7/T cells were maintained in Dulbecco’s Modified Eagle Medium (DMEM) containing 10% fetal bovine serum (FBS) and 1% penicillin and streptomycin (Gibco Life Technologies, Lofer, Austria). Primary human mammary epithelial cells (HUMECs) and its Ready Medium (Catalog No. 12752010) were purchased from Gibco, and human umbilical vein endothelial cells (HUVECs) were purchased from the National Infrastructure of Cell Line Resource^1^. The cells were maintained in DMEM supplemented with 10% FBS, 1% penicillin, 1% streptomycin (Gibco), 40 U/L insulin (Sigma, St. Louis, MO, United States), 40 U/mL heparin (Sigma), and 1% non-essential amino acids (Cyagen Biosciences, Santa Clara, CA, United States).

### Cell Number and Colony Formation Assay

After the indicated drug treatment, cell numbers were counted using trypan blue exclusion on a Cellometer Mini device (Nexcelom, Boston, MA, United States). Experiments were performed in triplicate. For the colony formation assay, cells at a density of 1 × 10^3^ cells/well were seeded into 6-well plates. After cell attachment, paclitaxel or ADQ was added to the wells alone or in combination for 4 h. Then the cells were cultured with fresh medium for 2 weeks. The colonies were fixed in 4% paraformaldehyde, stained with Coomassie Blue, photographed, and counted under a microscope.

### Flow Cytometry Analysis

For the drug efflux assay, MDA-MB-231 or MCF-7 cells were pretreated with ADQ for 24 h, followed by incubation with epirubicin for 60 min at 37°C. After dug exposure and washing, the cells were released in drug-free medium for 90 min and harvested for flow cytometry analysis. For cell cycle analysis, cells were fixed in ice-cold 70% ethanol at -20°C overnight. Then cells were washed with phosphate-buffered saline, stained with 50 mg/mL propidium iodide (Sigma), and dissolved in 100 mg/L RNase A (Sigma). For apoptosis analysis, the cells were stained with the Annexin V-FITC Apoptosis Staining/Detection Kit (BD Biosciences, San Jose, CA, United States). All flow cytometry analyses were conducted with FACSAria SORP (BD Biosciences) and analyzed by Modifit LT or FlowJo software.

### Immunofluorescence Analysis and Hoechst 33258 Staining

For measurements of phosphorylated histone *p*-H2AX expression, cells were incubated with 4% paraformaldehyde and 0.2% triton X-100 for 10 min. Following blocking in goat serum for 60 min, the samples were co-incubated with primary antibodies against *p*-H2AX (ABclonal Technology, Boston, MA, United States) at 4°C overnight and subsequently labeled with fluorescence-conjugated secondary antibodies for 2 h at room temperature. Then DAPI was applied for nuclear staining, and the signals were detected by fluorescence microscopy (TS2R; Nikon, Tokyo, Japan). With regard to Hoechst 33528 detection, cells were seeded at 60–70% confluency in 6-well plates, and then treated with the indicated drug for 48 h. Then Hoechst 33258 staining was conducted according to the manufacturer’s instructions.

### Establishment of the Herb–Ingredient–Target Interaction

The chemical ingredients were collected from TCM databases including the TCMSP Database^2^ the TCMID^3^, and the BATMAN-TCM^4^. The ingredients were screened according to drug likeness (DL) and OB values, and the ingredients were retained if DL ≥ 0.18 and OB ≥ 30, a criterion suggested by the TCMSP database. The ingredient–target networks were constructed for these herbs using Cytoscape software (version 3.2.1).

### Gene Ontology and Pathway Enrichment Analysis

Gene expression data were retrieved from the NCBI GEO database^5^, and then analyzed with the GEO2R online analysis tool^6^. The dataset GSE41112 includes 24 tumors of breast cancer patients with chemotherapy and 37 without chemotherapy, and the GSE87455 dataset includes human breast cancer samples with “no treatment” (*n* = 122) and “chemo only” (*n* = 83) groups. The DEGs were screened with *P* ≤ 0.05 and fold control (FC) ≥ 1.5 criteria, delivered to the Search Tool for the Retrieval of Interacting Genes/Protines (STRING) database to evaluate the PPI information, and also submitted to the DAVID^7^ for enrichment analysis. The significant enrichment analysis of DEGs was assessed based on the GO and KEGG^8^.

### Plasmids and Small Interfering RNA Construction and Transfection

The pcDNA 3.1(+)-CAV1 was provided by Vigene Company (Jinan, China) and transfected into cells using Lipofectamine 2000 (Invitrogen, Carlsbad, CA, United States). After 24 h, the transfected cells were passaged and selected for 2 weeks with 10 μg/mL puromycin (Invitrogen). Pooled populations of positive cells were used for subsequent experiments. Negative control cell lines were generated by transfecting cells with scrambled plasmids. The small interfering RNAs (siRNAs) targeting CAV1 or scrambled siRNAs were purchased from Invitrogen (Carlsbad) and transfected using the X-tremeGENE siRNA transfection reagent (Roche Diagnostics, Indianapolis, IN, United States) according to the manufacturer’s instructions.

### Western Blotting

To determine the protein concentration, cells were lysed in RIPA buffer (Sigma) containing a protease inhibitor mixture (Roche Diagnostics). The protein concentration was measured with the bicinchoninic acid assay (Thermo Fisher Scientific, Bonn, Germany). Quantified protein lysates (15 μg) were subjected to sodium dodecyl sulfate polyacrylamide gel electrophoresis and resolved on 12% polyacrylamide gels. Then the proteins were transferred onto a PVDF membrane (GE Healthcare, Freiburg, Germany). The membrane was probed with primary antibodies including CAV1, p21, cyclin B1, cleaved poly (ADP-ribose) polymerase (PARP), Bcl2-associated X protein (BAX), B-cell lymphoma 2 (Bcl-2), p53, and phosphorylated p53 (p-p52, ser 15), and β-actin (Cell Signaling Technology, Beverly, MA, United States) at 4°C overnight. After washing three times with Tris-buffered saline and 0.05% Tween-20, the membrane was incubated with secondary anti-rabbit or anti-mouse antibodies for 2 h at room temperature. The signals were visualized using the ECL Advance Western Blotting Detection Reagent (GE Healthcare) and quantified with FlowJo software.

### Breast Cancer Mice Models and Drug Treatment

All animal procedures were performed in accordance with institutional guidelines for the care and use of laboratory animals approved by the Animal Care and Use Committee of Guangzhou University of Chinese Medicine and the National Institutes of Health guide for the care and use of laboratory animals. The mouse mammary tumor virus (MMTV)-PyMT mouse model of breast cancer spontaneously develops 100% multiple and luminal-like breast tumors from normal mammary epitheliums by 8–12 weeks, similar to the pathological processes and characteristics in human breast cancer (Lin et al., 2003; Wang et al., 2013). In this study, 9-week-old MMTV-PyMT mice were randomly divided into four groups, and treated with saline (Ctrl group), 10 mg/kg paclitaxel (paclitaxel group), 100 mg/kg ADQ (ADQ group), or 10 mg/kg paclitaxel plus 100 mg/kg ADQ (paclitaxel + ADQ group) for 26 days (*n* = 6 mice, total of 60 glands), respectively. Paclitaxel was given by intraperitoneal injection at 10 mg/kg every 3 days, and ADQ was given by oral gavage at 100 mg/kg once a day. The body weight and tumor volumes were recorded throughout the whole experimental period.

### Hematoxylin and Eosin Staining, Immunohistochemistry, and Terminal Deoxynucleotidyl Transferase dUTP Nick End Labeling

Hematoxylin and eosin staining and IHC were conducted according to the protocol provided by Wang et al. (2017a). The terminal dexynucleotidyl transferase dUTP nick end labeling (TUNEL) assay was conducted according to the manufacturer’s instructions (Catalog No. KGA7051; KeyGen Biotech, Nanjing, China).

### Statistical Analysis

The data are shown as the mean ± standard deviation. The two-tailed Student’s *t*-test or one-way analysis of variance was used to determine the significance of the data between groups. *P*-values less than 0.05 were considered statistical significant.

## Results

### ADQ Markedly Inhibits Growth in Paclitaxel-Sensitive and Paclitaxel-Resistant Human Breast Cancer Cells

**FIGURE 1:**
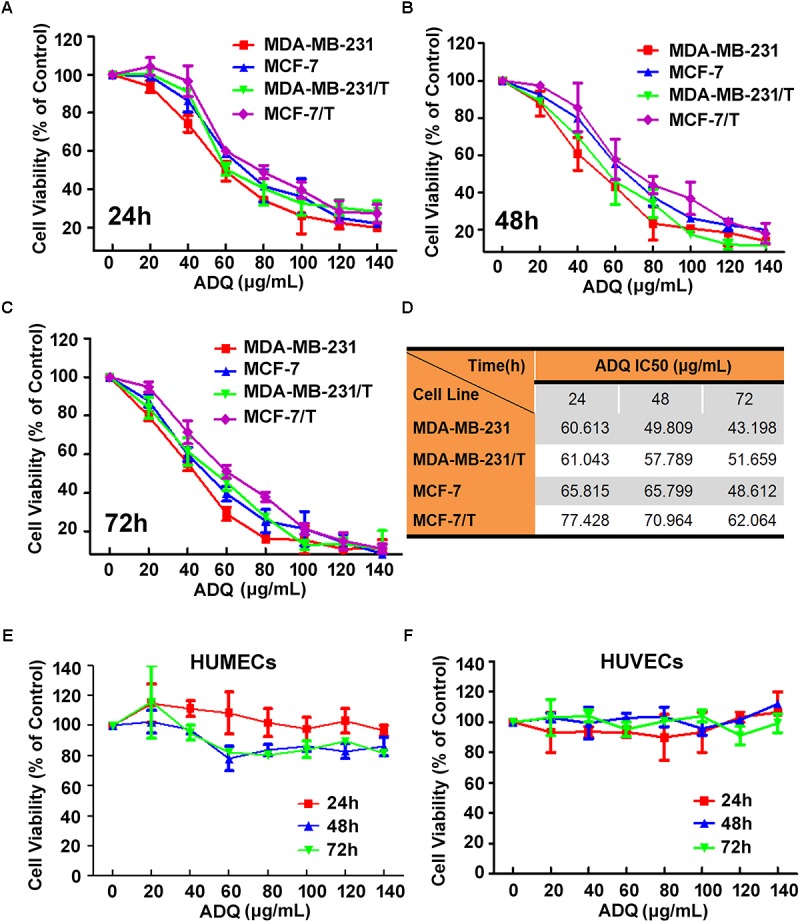
Effects of ADQ on cell proliferation in the paclitaxel-sensitive breast cancer cells and paclitaxel-resistant human breast cancer cells. **(A–C)** Exponentially growing cells of MDA-MB-231, MCF-7, MDA-MB-231/T, and MCF-7/T were treated with ADQ at the indicated concentrations (0–140 μg/mL) for 24, 48, and 72 h; **(D)** The IC50s of ADQ for MDA-MB-231, MDA-MB-231/T, MCF-7, and MCF-7/T cells; **(E,F)** Exponentially growing cells of HUMECs and HUVECs were treated with ADQ at the indicated concentrations (0–140 μg/mL) for 24, 48, and 72 h.

For quality control of ADQ, the chromatographic fingerprinting of ADQ was conducted and quantitative analysis of *p*-coumaric acid (peak 1), calycosin-7-glucoside (peak 2), liquiritin (peak 3), glycyrrhizic acid (peak 4), and curcumol (peak 5) in ADQ was compared among different batches (**Supplementary Figure S1**). Based on an established HPLC method, good linearity of five compounds was achieved with a correlation coefficient of *R^2^*≥ 0.9995 (**Supplementary Table S1**). The retention times of these compounds were determined to be 19.8, 23.9, 25.8, 50.7, and 69.5 min, respectively. The contents of the five compounds in the ADQ samples were also determined (**Supplementary Table S2**). We evaluated the influence of ADQ on the proliferation of breast cancer cell lines including MDA-MB-231 and MCF-7, as well as their derived paclitaxel-resistant cell lines MDA-MB-231/T and MCF-7/T. Significant inhibition of growth was observed in both parental and resistant cells at 24 h (**Figure 1A**), 48 h (**Figure 1B**), and 72 h (**Figure 1C**). IC_50_ of ADQ was shown in **Figure 1D** for the indicated cell lines. Specifically, the IC_50_ values of ADQ at 48 h for MDA-MB-231, MDA-MB-231/T, MCF-7, and MCF-7/T were 49.809, 57.789, 65.799, and 70.964 μg/mL, respectively, suggesting that ADQ had similar suppressive effects on both sensitive and resistant breast cancer cells. To determine the cytotoxic effects of ADQ on normal cells, we also investigated its effects on HUMECs and HUVECs, and found that ADQ did not have cytotoxic inhibitory effects on both types of normal cells (**Figures 1E,F**). These findings indicated that ADQ exerted selective toxic effects on breast cancer cells.

### ADQ Significantly Enhances the Chemosensitivity of Breast Cancer Cells

**FIGURE 2:**
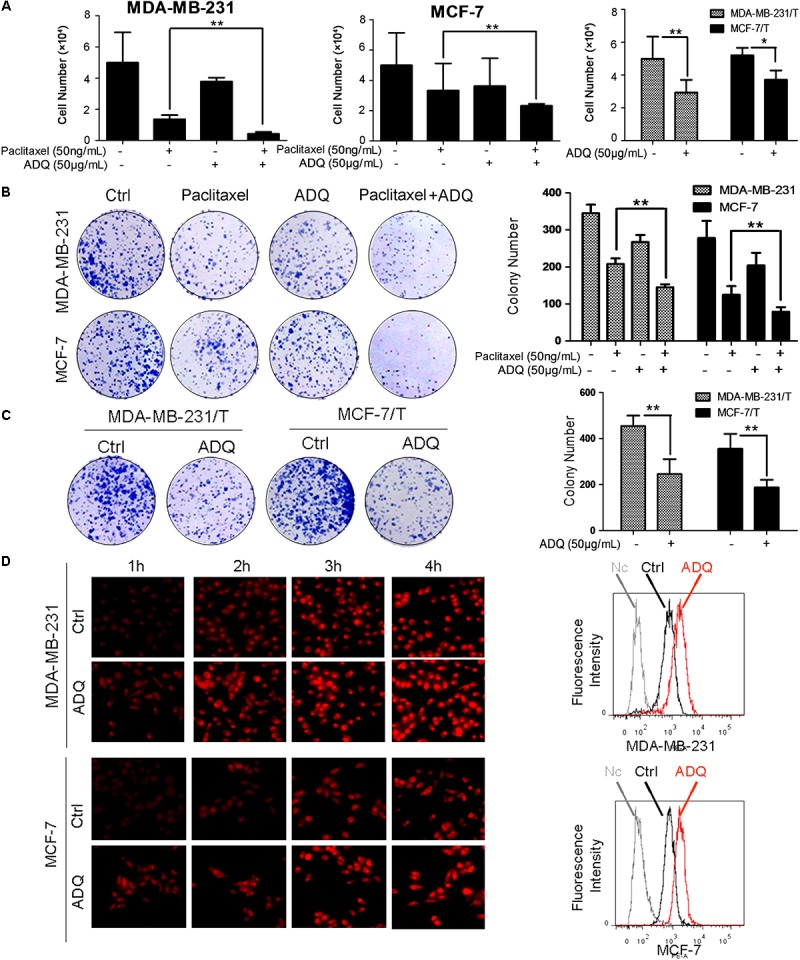
ADQ significantly enhances the chemosensitivity of breast cancer cells. **(A)** The synergistic effects of ADQ (50 μg/mL) with paclitaxel (50 ng/mL) on MDA-MB-231 and MCF-7 cells, and the inhibitory effects of ADQ (50 μg/mL) on MDA-MB-231/T and MCF-7/T cells were studied by the cell counting assay after 48h treatment (^∗^*P* < 0.05 *v.s.* control, ^∗∗^*P* < 0.01 *v.s.* control, values represented as the mean ± SD, *n* = 3); **(B)** Colony formation assay for evaluating the long-term inhibitory effects of ADQ (50 μg/mL) with or without paclitaxel (50 ng/mL) on MDA-MB-231 and MCF-7 cells; **(C)** Colony formation assay for evaluating the long-term inhibitory effects of ADQ (50 μg/mL) on MDA-MB-231/T and MCF-7/T cells (^∗^*P* < 0.05 *v.s.* control, ^∗∗^*P* < 0.01 *v.s.* control, values represented as the mean ± SD, *n* = 3); **(D)** the drug efflux assay revealed that ADQ (50 μg/mL) could enhance the epirubicin intake into breast cancer cells, as shown by increased fluorescence intensity of epirubicin in ADQ-treated cells by flow cytometry analysis.

To determine the synergistic activities of ADQ with paclitaxel in breast cancer, MDA-MB-231 and MCF-7 cells were treated with ADQ and paclitaxel for 48 h. As presented in **Figure 1** and **Supplementary Figure S2**, the tested concentrations of taxol and ADQ were set according to their IC_50_ at 48 h. ADQ significantly enhanced paclitaxel-induced death in MDA-MB-231 and MCF-7 cells. Interestingly, ADQ administration at 50 μg/mL also caused a clear reduction in the number of resistant breast cancer cells (**Figure 2A**). We also evaluated the long-term inhibitory effects of ADQ on the colony formation capabilities of breast cancer cells with or without paclitaxel. The results showed that the combination group exhibited a substantial reduction in the colony numbers of parental MDA-MB-231 and MCF-7 cells (**Figure 2B**). In addition, the growth of paclitaxel-resistant MDA-MB-231/T and MCF-7/T cells was greatly suppressed in the presence of ADQ (**Figure 2C**). The drug efflux assay revealed that ADQ could enhance epirubicin influx into breast cancer cells, as shown by the increased fluorescence intensity of epirubicin in ADQ-treated cells (**Figure 2D**). These results indicated that ADQ could chemosensitize both paclitaxel-sensitive and paclitaxel-resistant human breast cancer cells.

### ADQ Induced Breast Cancer Cell Cycle Arrest at the G2/M Checkpoint

**FIGURE 3:**
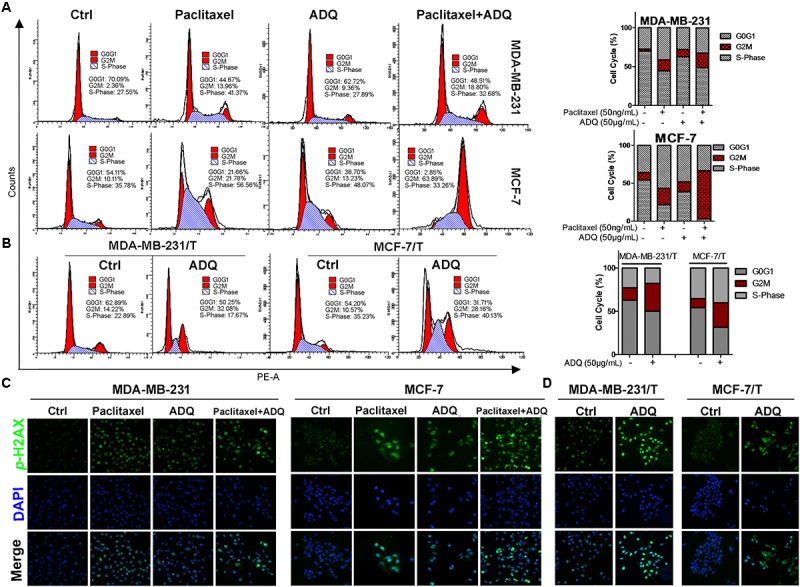
ADQ and paclitaxel induce breast cancer cell cycle arrest at the G2/M phase. Representative cell cycle analysis of **(A)** the MDA-MB-231 and MCF-7 cells treated with ADQ (50 μg/ml) and/or paclitaxel (50 ng/mL), as well as **(B)** the MDA-MB-231/T and MCF-7/T cells treated with ADQ (50 μg/mL) for 48 h using flow cytometry. The length of each cell cycle phase was calculated; The immunofluorescence assay was performed to examine *p*-H2A.X **(C)** in the MDA-MB-231 and MCF-7 cells treated with ADQ (50 μg/mL) and/or paclitaxel (50 ng/mL), as well as **(D)** in the MDA-MB-231/T and MCF-7/T cells treated with ADQ (50 μg/mL) for 48 h.

Uncontrolled cell mitosis represents one of the hallmarks of cancer. Thus, we used PI staining to examine the effects of ADQ on the cell cycle distribution of both paclitaxel-sensitive and paclitaxel-resistant cells. As labeled in **Figure 3A**, the flow cytometry results revealed that paclitaxel or ADQ alone could induce G2/M checkpoint arrest in both breast cancer cell lines. In addition, ADQ and paclitaxel combination increased G2/M arrest by 26% in MDA-MB-231 cells and by 66% in MCF-7 cells (**Figure 3A**). Furthermore, the G2/M population of MDA-MB-231/T and MCF-7/T cells was also arrested by ADQ with a 56 and 63% increase, respectively (**Figure 3B**). Previous studies have shown that perturbation of the G2/M transition was largely due to DNA damage, and *p*-H2AX could be a marker for monitoring DNA damage (Sancar et al., 2004). The immunofluorescence results showed that *p*-H2AX intensity was significantly elevated following paclitaxel or ADQ treatment in both breast cancer cell lines. Consistent with the cell cycle findings, ADQ synergistically interacted with paclitaxel to enhance *p*-H2AX expression in both breast cancer cell lines (**Figure 3C**). Furthermore, the expression of *p*-H2AX in MDA-MB-231/T and MCF-7/T cells was also significantly enhanced following ADQ administration, indicating that the chemosensitizing effects of ADQ might be attributed to DNA damage-induced G2/M checkpoint arrest (**Figure 3D**).

### ADQ Augmented Paclitaxel-Induced Apoptosis in Breast Cancer Cells

**FIGURE 4:**
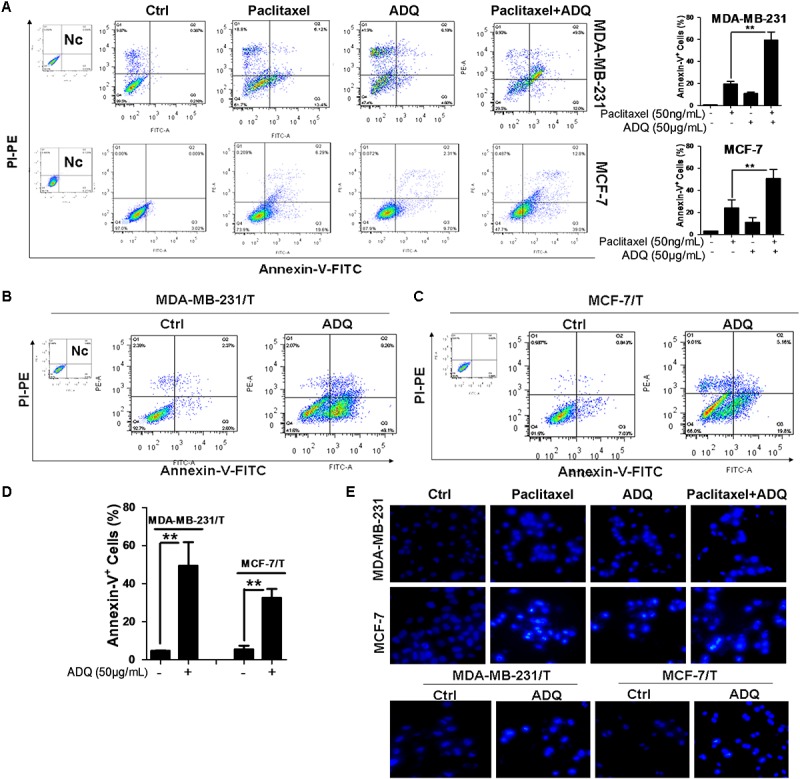
ADQ augments paclitaxel-induced apoptosis in breast cancer cells. Representative apoptosis analysis of **(A)** the MDA-MB-231 and MCF-7 cells treated with ADQ (50 μg/mL) and/or paclitaxel (50 ng/mL), as well as **(B–D)** the MDA-MB-231/T and MCF-7/T cells treated with ADQ (50 μg/mL) for 48h using flow cytometry (^∗^*P* < 0.05 *v.s.* control, ^∗∗^*P* < 0.01 *v.s.* control, values represented as the mean ± SD, *n* = 3); **(E)** Hoechst 33258 staining showed typical apoptotic morphology changes of cells after indicated treatment.

Apoptosis is another important mechanism that causes cell death induced by chemotherapy drugs (Johnstone et al., 2002; Li et al., 2010). To determine if ADQ could synergistically aggravate paclitaxel-induced apoptosis, Annexin V/PI staining was applied to detect the apoptotic events. As shown in **Figure 4A**, the percentage of early and late apoptotic events in MDA-MB-231 and MCF-7 cells reached about 20 and 25%, respectively, after exposure to 50 ng/mL paclitaxel. Interestingly, when ADQ was administrated with paclitaxel concurrently, the percentage of MDA-MB-231 and MCF-7 apoptotic cells was increased to approximately 60% and 51%, respectively. Notably, ADQ was also capable of inducing apoptosis in paclitaxel-resistant breast cancer cells. The percentage of apoptotic cells in MDA-MB-231/T reached 45% following ADQ treatment after 48 h, and MCF-7/T cells reached 30% (**Figures 4B–D**). Based on flow cytometry results, Hoechst 33258 staining was utilized to observe the morphological changes of apoptotic cells by fluorescence imaging (Zhang et al., 2010). In paclitaxel-sensitive breast cancer cells, ADQ synergistically enhanced Hoechst 33258 staining intensity induced by paclitaxel in both MDA-MB-231 and MCF-7 cells. In paclitaxel-resistant cell models, typical morphological characteristics of apoptosis, such as chromatin condensation and cell pyknosis, were more easily observed following ADQ treatment (**Figure 4E**). Together, these findings confirmed that ADQ promoted paclitaxel-induced apoptosis in breast cancer cells.

### Network Pharmacology Analysis of ADQ

**FIGURE 5:**
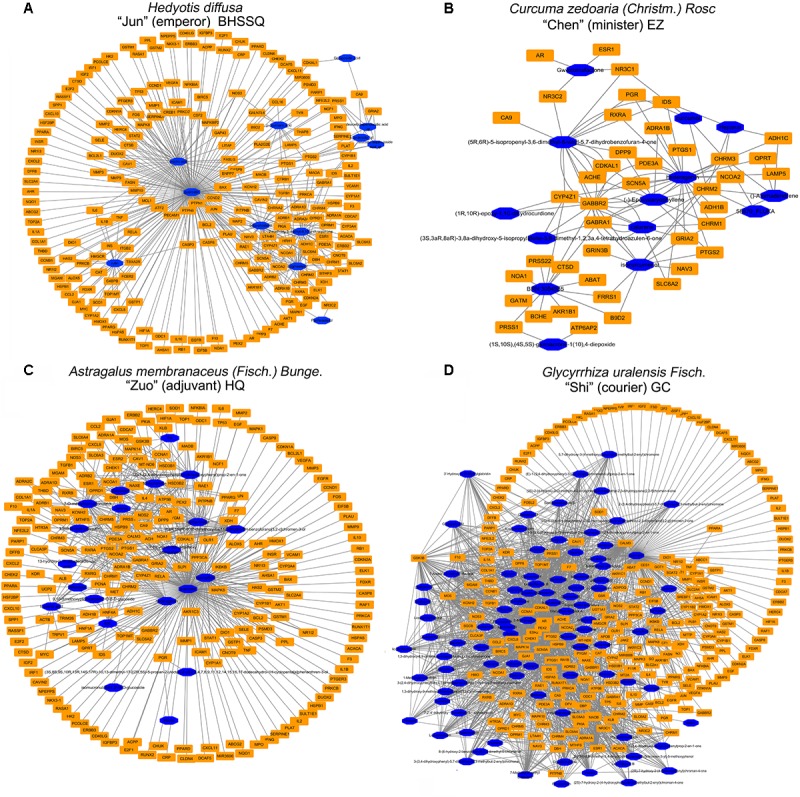
The ingredient-target network of single herb in ADQ. **(A)** BHSSC; **(B)** EZ; **(C)** HQ; and **(D)** GC. The blue diamond nodes represent ingredients, and the orange rectangle nodes represent targets.

**FIGURE 6:**
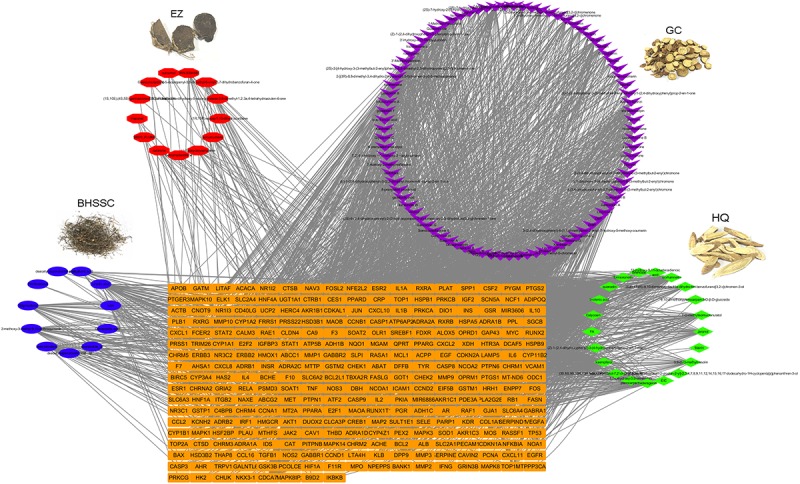
The ingredient-target network of ADQ. The compound-target network was constructed by linking the herbs, candidate compounds and all their potential targets. The nodes represent ingredients of herbs (blue for BHSSC, red for EZ, purple for GC, and green for HQ), and the orange rectangle nodes represent the shared targets of these four herbs.

ADQ consists of four herbs including BHSSC, EZ, HQ, and GC. To establish the ingredient–target network of ADQ, we screened candidate compounds for (OB ≥ 30%) and DL (DL ≥ 0.1) in each herb. There were 12 compounds in BHSSC targeting 225 genes (**Figure 5A**), 14 compounds in EZ targeting 41 genes (**Figure 5B**), 21 compounds in HQ targeting 222 genes (**Figure 5C**), and 94 compounds in GC targeting 241 genes (**Figure 5D**). The “candidate active compounds” are listed in **Supplementary Table S3**. Overall, our results showed that ADQ yielded 132 candidate compounds and 297 potential targets after eliminating all duplicates (**Figure 6**). Specifically, the network included 429 nodes and 2874 ingredient–target interactions, of which 132 candidate compounds had a median of 10 target correlations, suggesting the existence of complex correlations among different compounds and targets.

### Establishment of the Compound–Target–Disease Network of ADQ

**FIGURE 7:**
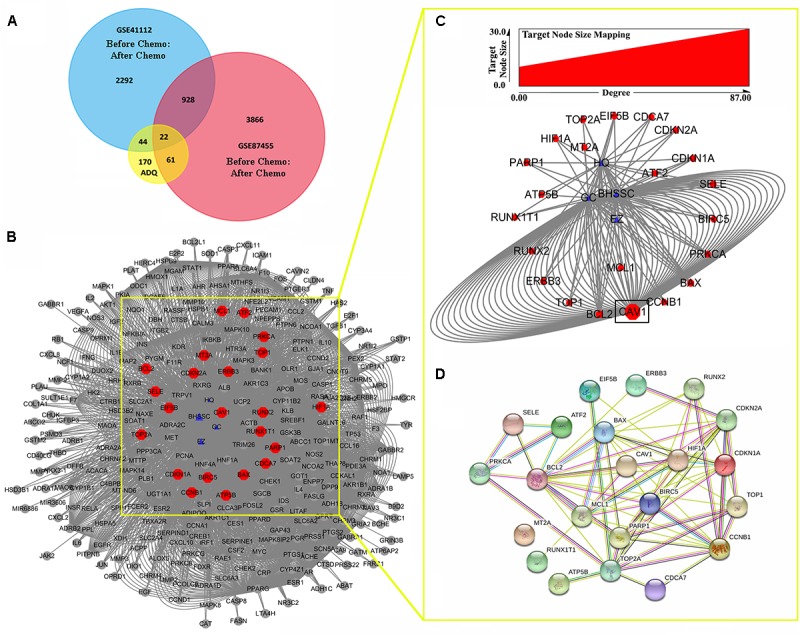
Potential genes involved in the chemosensitizing effects of ADQ against breast cancer. **(A,B)** The venn analysis showed the crosstalk of DEGs in ADQ and datasets GSE5764/GSE87455 of breast cancer patients before and after chemotherapy; **(C)** CAV1 was the key target with the largest node size according to “Degree” in the node size mapping; **(D)** The PPI of hub targets involved in the chemosensitizing effects of ADQ. The blue triangle nodes represent ADQ herbs, and the red rectangle nodes represent the 22 hub targets.

To determine the pharmacological mechanisms of ADQ against chemoresistance, the DEGs of breast cancer patients before and after chemotherapy were extracted from GSE41112 and GSE87455 microarray sets. A total of 3286 genes in GSE5764 and 4877 genes in GSE87455 were identified using the GEO2R analysis tool (*P* ≤ 0.05, FC ≥ 1.5). The Venn diagram analysis showed that ADQ shared 22 putative targets with the two datasets (**Figure 7A**). These hub genes included CAV1, HIF1A, CCNB1, BAX, BCL2, PARP1, ERBB3, MCL1, PRKCA, CDCA7, CDKN1A, CDKN2A, TOP1, TOP2A, SELE, ATP5B, RUNX2, BIRC5, ATF2, RUNX1T1, MT2A, and EIF5B (**Figure 7B**), among which CAV1 was the key target with the largest node size according to “Degree” in the node size mapping (**Figure 7C**). We further extracted the 22 significant targets to construct the PPI containing 22 nodes and 71 edges based on the STRING database, and the PPI enrichment *P*-value of these hub genes was 2.22 × 10^-16^, indicating that they were at least partially biologically connected (**Figure 7D**). All of these findings suggested that CAV1 might be one of the the most likely mechanisms affecting ADQ regulation network.

**FIGURE 8:**
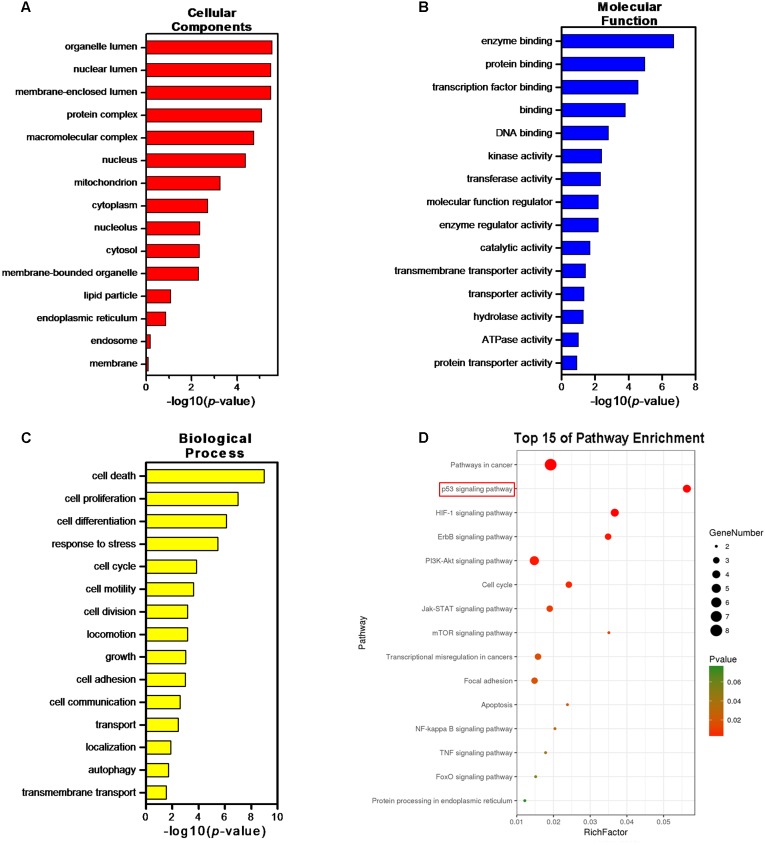
Gene Ontology (GO) and Pathway enrichment analysis of the crosstalk targets. GO terms analysis of the 22 hub genes containing 3 aspects including **(A)** cellular components, **(B)** molecular function, and **(C)** biological process. **(D)** Pathway enrichment analysis of the 22 hub genes.

**FIGURE 9:**
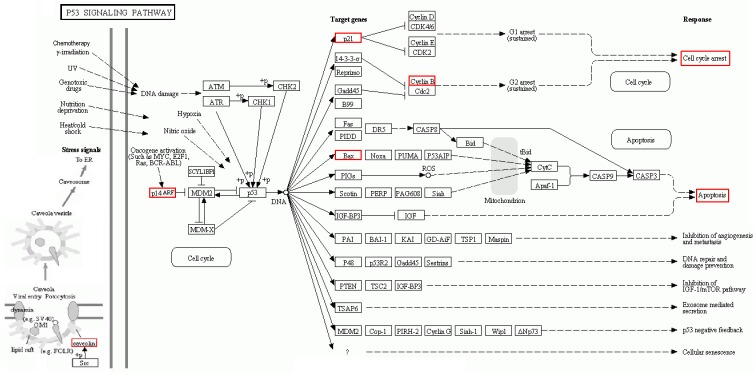
KEGG pathway suggested that various targets in p53 signaling were tightly associated with ADQ pharmacological action. The red rectangle nodes represent the most significant genes or biological pathways associated with ADQ pharmaoclogical action (*P* = 0.00003896097).

Thus, we continued to identify candidate targets by setting all human genes as background, and using GO and pathway enrichment analysis. GO classified function into categories of cellular components, molecular function, and biological process. The top three enrichments in the cellular components category were organelle lumen, nuclear lumen, and membrane-enclosed lumen (**Figure 8A**); in the molecular function category were enzyme binding, protein binding, and transcription factor binding (**Figure 8B**); and in the biological process category were cell death, cell proliferation, and cell differentiation (**Figure 8C**). In **Figure 8D**, KEGG analysis (*P* < 0.05) indicated that multiple cancer-related pathways were significantly involved in the mechanisms of ADQ including focal adhesion, apoptosis, cell cycle, p53, HIF-1, ErbB, phosphoinositide 3-kinase (PI3K)/Akt, Janus kinase/signal transducer and activator of transcription, mammalian target of rapamycin, NF-kappa B, and tumor necrosis factor signaling pathways. Notably, It was various targets in p53 signaling were tightly associated with ADQ pharmacological action (*P* = 0.00003896097, red rectangle, **Figure 8D**). P53 signaling can be stimulated by a number of stress signals including DNA damage, oxidative stress, and activated oncogenes, consequently leading to cell cycle arrest and apoptosis (Mello and Attardi, 2017). Meanwhile, accumulating evidence has demonstrated that CAV1, the leading hub target of ADQ, is correlated with various stressors including chemotherapy, radiotherapy, fluid shear, oxidative stress, and ultraviolet exposure (Wang et al., 2015c). This novel stress response protein plays significant roles in modulating cell survival, proliferation, and apoptosis (Wang et al., 2017d). In **Figure 9**, we postulated that CAV1 might be activated in response to ADQ treatment, subsequently influencing the cell cycle regulatory proteins p21 and cyclin B1, and apoptotic markers such as BAX.

### Validation of CAV1 as a Major Chemosensitizing Target of ADQ in Breast Cancer

**FIGURE 10:**
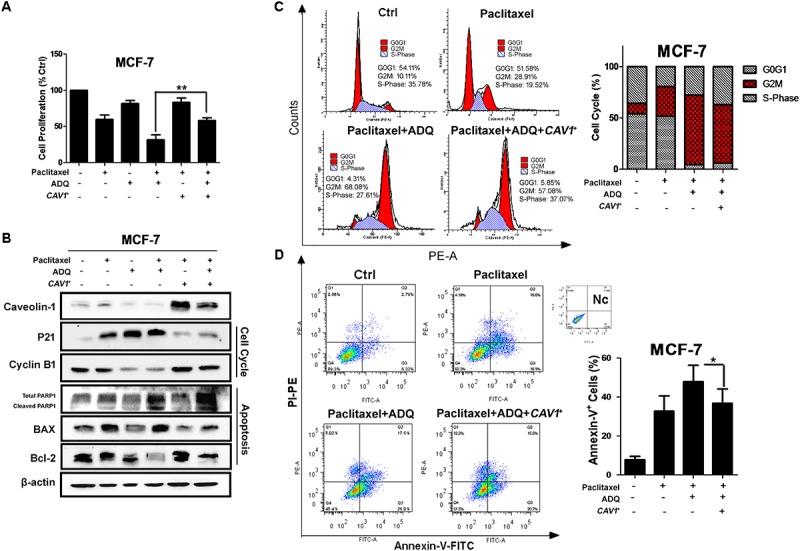
*CAV1* overexpression abrogated the anti-cancer and chemosensitizing effects induced by ADQ on breast cancer cells. *CAV1*-deficient MCF-7 cells were transfected with caveolin-1 recombinant plasmids and treated with ADQ (50 μg/ml) and/or paclitaxel (50 ng/ml) for 48 h. **(A)** The cell counting assay revealed that *CAV1* overexpression obviously abrogated the synergistic effects of ADQ with paclitaxel in suppressing breast cancer growth (^∗∗^*P* < 0.01 *v.s.* control, values represented as the mean ± SD, *n* = 3); **(B)** The expressions of CAV1, p21, cyclinB1, PARP1, BAX and Bcl-2 using western blotting analysis after indicated treatment; **(C)** Representative cell cycle demonstrated CAV1 overexpression relieved the G2/M arresting effects of ADQ from 68.08 to 57.08% in MCF-7 cells using flow cytometry. The length of each cell cycle phase was calculated; **(D)** Representative apoptosis analysis indicated *CAV1* overexpression reduced the apoptotic events induced by paclitaxel plus with ADQ treatment from 47.890 to 36.780% using flow cytometry (^∗^*P* < 0.05 *v.s.* control, values represented as the mean ± SD, *n* = 3).

We continued to validate whether the chemosensitizing activity of ADQ was *CAV1*-dependent. Among the multiple breast cancer cell lines tested, ZR75–1, SKBR3, and MCF7 had no or low CAV1 expression, whereas MDA-MB-231 and MDA-MB-436 had strong CAV1 expression (Shi et al., 2015). In this study, we detected the expression of CAV1 among a series of breast cancer and normal mammary cell lines. The results showed that CAV1 expression was significantly downregulated in the MDA-MB-231 and MCF-7 breast cancer cells, and was enhanced in their taxol-resistant counterparts (**Supplementary Figure S3**). This phenomenon was consistent with the oncogenic and tumor suppressor roles of CAV1 in breast cancer development (Wang et al., 2015c). To directly examine whether CAV1 was critical for ADQ action, we elevated CAV1 levels by transfecting recombinant CAV1 plasmid in *CAV1*-deficient MCF-7 cells, and decreased its expression with si*CAV1* in *CAV1*-expressing MDA-MB-231 cells. CAV1 expression in the gene-modified cells was confirmed by western blot analysis (**Supplementary Figure S4A**). Then we determined if CAV1 overexpression abrogated the anti-cancer and chemosensitizing effects induced by ADQ in breast cancer cells. As shown in **Figure 10A**, ADQ synergistically interacted with paclitaxel to suppress MCF-7 cell proliferation, whereas CAV1 overexpression obviously abrogated the synergistic effects of ADQ with paclitaxel in suppressing breast cancer growth (*p* ≤ 0.01). To determine if CAV1 signaling was involved in ADQ chemosensitizing activity, the indicated proteins were analyzed (**Figure 10B**). Western blot analysis revealed that ADQ alone could inhibit CAV1 expression, accompanied by the increased expression of p21 and decreased expression of cyclin B1. In addition, the expression of cleaved PARP1 and BAX were enhanced by ADQ, whereas Bcl-2 expression was decreased following ADQ treatment. Notably, CAV1 overexpression resulted in decreased p21 and increased cyclin B1 expression. Furthermore, CAV1 overexpression inhibited apoptosis, as shown by the decreased expression of cleaved PARP1 and BAX and increased Bcl-2 expression. Cell cycle analysis further revealed that CAV1 overexpression relieved the G2/M arresting effects of ADQ from 68.08 to 57.08% in MCF-7 cells (**Figure 10C**). CAV1 overexpression also significantly reduced the apoptotic events induced by paclitaxel with ADQ treatment from 47.89 to 36.78% (**Figure 10D**). These data showed that ADQ inhibited CAV1 to improve breast cancer chemosensitivity.

**FIGURE 11:**
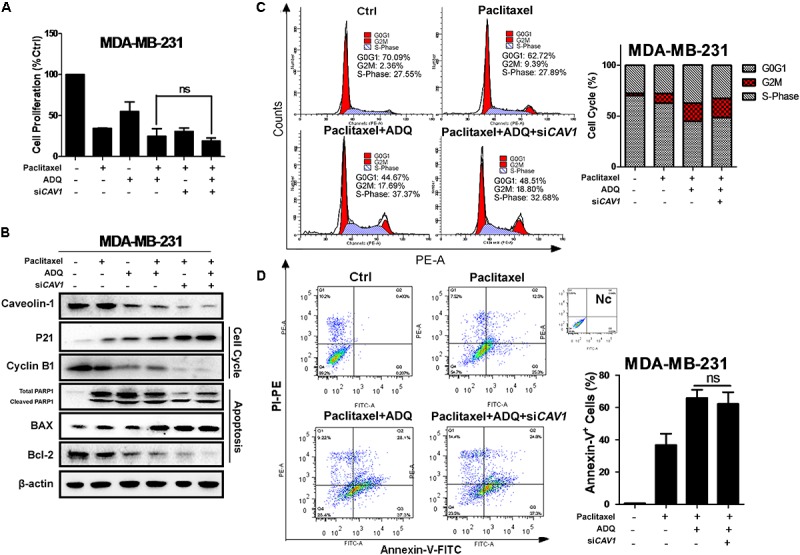
*CAV1* knockdown exerted no additive anti-cancer and chemosensitivity effects induced by ADQ on breast cancer cells. *CAV1*-expressing MDA-MB-231 cell were exposed to si*CAV1* for 24 h and treated with or without ADQ (50 μg/ml) and/or paclitaxel (50 ng/ml) for additional 24 h. **(A)** the cell counting assay demonstrated that si*CAV1* administration did not further increase the chemosensitivity of ADQ; **(B)** The expressions of CAV1, p21, cyclinB1, PARP1, BAX and Bcl-2 using western blotting analysis after indicated treatment; si*CAV1* did not significantly enhance the chemosensitivity of ADQ by altering **(C)** cell cycle distribution and **(D)** apoptotic population with flow cytometry assay.

**FIGURE 12:**
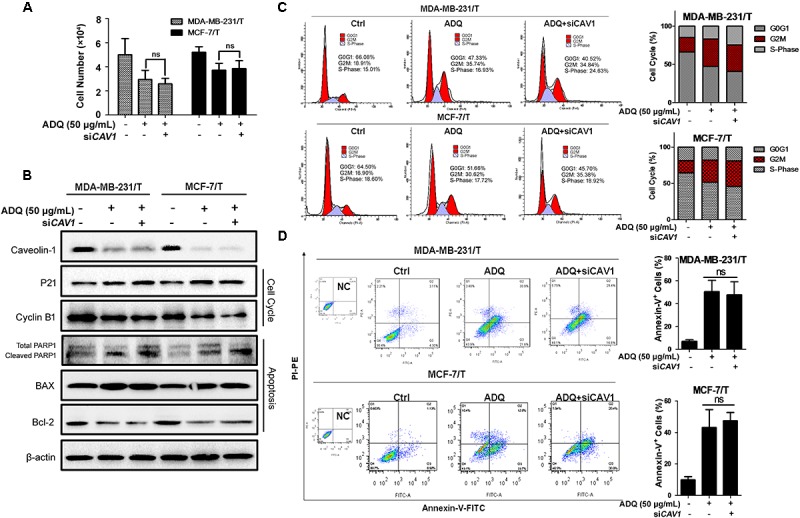
*CAV1* knockdown exerted no additive effects induced by ADQ on Reversal of multidrug-resistance in paclitaxel-resistant breast cancer cells. MDA-MB-231/T and MCF-7/T cell were exposed to si*CAV1* for 24 h and treated with or without ADQ (50 μg/ml) for additional 24 h, respectively. **(A)** the cell counting assay demonstrated that si*CAV1* administration did not further increase the inhibition ratio when ADQ was added; **(B)** The expressions of CAV1, p21, cyclinB1, PARP1, BAX and Bcl-2 using western blotting analysis after indicated treatment; si*CAV1* did not significantly enhance the suppressive activities of ADQ by altering **(C)** cell cycle distribution and **(D)** apoptotic population with flow cytometry assay.

In *CAV1*^high^ MDA-MB-231 cells, CAV1 knockdown did not significantly aggravate the inhibitory ability of ADQ in the presence of paclitaxel, suggesting that ADQ might target CAV1 to chemosensitize breast cancer (**Figure 11A**). Similarly, si*CAV1* did not significantly change the expression levels of p21, cyclin B1, PARP1, BAX, and Bcl-2 when administrated together with paclitaxel and ADQ (**Figure 11B**), nor were significant changes observed in cell cycle distribution and apoptotic cells (**Figures 11C,D**). Furthermore, MDA-MB-231/T and MCF-7/T showed strong CAV1 expression compared with their parental cell lines (**Supplementary Figure S4B**). In paclitaxel-resistant breast cancer cell models, both CAV1 silencing and ADQ treatment resulted in the significant suppression of cell proliferation, but CAV1 silencing did not increase the effects of ADQ, namely inhibition of proliferation, cell cycle arrest, or apoptosis induction in MDA-MB-231/T and MCF-7/T cells (**Figures 12A–D**). These findings demonstrated that ADQ could target CAV1 to induce cell cycle arrest and apoptosis, thereby chemosensitizing paclitaxel-resistant breast cancer cells.

### ADQ Enhanced *in vivo* Paclitaxel Chemosensitivity on Breast Cancer

**FIGURE 13:**
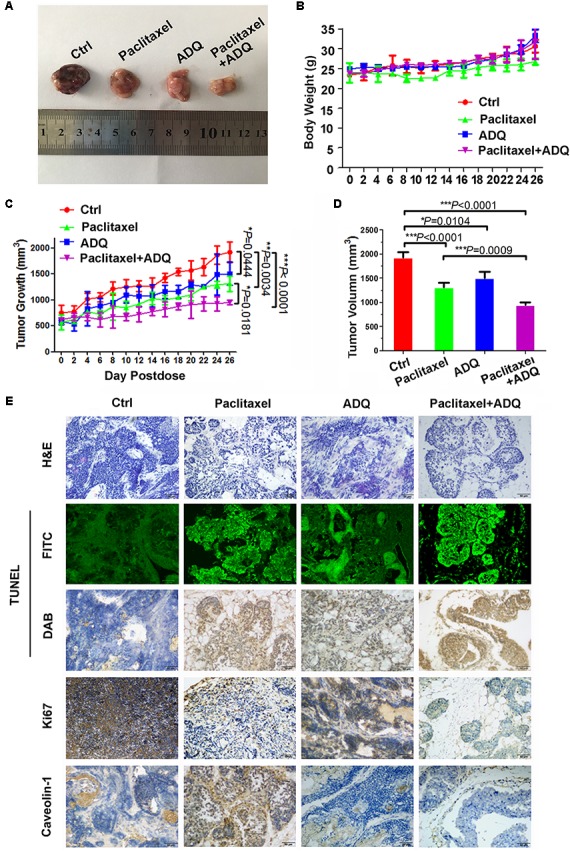
ADQ enhanced *in vivo* paclitaxel chemosensitivity on breast cancer. 9-week-old MMTV-PyMT mice were randomly divided into four groups, and were treated with vehicle (Ctrl group), 10 mg/kg paclitaxel (Paclitaxel group), 100 mg/kg ADQ (ADQ group), or 10mg/kg paclitaxel plus 100 mg/kg ADQ (Paclitaxel + ADQ group) according to designated treatment schedule. **(A)** Representative images of tumors dissected, **(B)** Body weight, and **(C,D)** Tumor volumes, and **(E)** H&E staining, TUNEL detection, and IHC detection of Ki67 and CAV1 expressions from the indicated groups (*n* = 6 mice, total of 60 glands, ^∗^*P* < 0.05, ^∗∗^*P* < 0.01, ^∗∗∗^*P* < 0.0001 *v.s.* control).

We finally evaluated the *in vivo* efficacy of ADQ on breast cancer with the MMTV-PyMT transgenic mouse model, which spontaneously develops 100% multiple and luminal-like breast tumors from normal mammary epitheliums by 8–12 weeks, similar to the pathological processes and characteristics in human breast cancer (Lin et al., 2003; Wang et al., 2013). Therefore, it is an appropriate preclinical model for investigating the chemosensitivity effects of ADQ with paclitaxel (**Figure 13A**). The results of the *in vivo* experiments revealed that ADQ alone could significantly inhibit breast tumor growth (^∗^*P* = 0.0444, **Figure 13C**) and reduce tumor volume (^∗^*P* = 0.0104, **Figure 13D**), while with little influence on mouse body weight (**Figure 13B**). Meanwhile, ADQ alone caused an increase in the apoptotic ratio and a decrease in ki67 expression, further demonstrating its tumor suppressive functions (**Figure 13E**). The results also revealed that the combination of paclitaxel and ADQ exerted stronger inhibitory effects on overall tumor growth than paclitaxel alone (**Figure 13C**). Notably, ADQ did not lead to significant weight loss throughout the experiment, whereas a slight reduction of body weight was found in the paclitaxel-treated mice (**Figure 13B**). Furthermore, both H&E staining and the TUNEL assay revealed that the synergistic use of paclitaxel and ADQ resulted in significant increases of apoptosis in tumor tissues, accompanied by the decreased expression of Ki67 and CAV1 (**Figure 13E**). These results indicate that ADQ is a potential adjuvant drug for breast cancer treatment with good safety.

## Discussion

With increasing attention being paid to multi-target strategies for cancer treatment, TCM has become a valuable resource. In this study, we investigated the anti-cancer and chemosensitizing functions of ADQ extracts in breast cancer. Our findings revealed that ADQ significantly inhibited the growth of breast cancer cells by cell cycle arrest and apoptosis induction. In particular, ADQ did not cause cytotoxic effects in normal cells. Similar findings have also been shown for other herbs including Huangqi, Ginseng, Banzhilian, Huachansu injection, TJ-48, Shenqi fuzheng injection, and Kanglaite injection (Qi et al., 2015). Notably, the four-herb formula PHY906 was developed as an adjuvant therapy with chemosensitizing effects. Currently, three phase I and one phase II clinical trial on PHY906 have been completed in the United States. In these clinical studies, PHY906 administration led to the reduction of chemotherapy-associated side effects in patients with metastatic colorectal cancer (PHY906+CPT-11/5FU/LV) and advanced pancreatic cancer (PHY906+capecitabine) (Lam et al., 2015). Omics studies have demonstrated that PHY906 can inhibit colon cancer growth by modulating cell apoptosis by intervening interferon-gamma production and responses to steroid hormone stimulus. Our study also demonstrated that ADQ could increase breast cancer chemosensitivity to paclitaxel by increasing breast cancer cell apoptosis and cell cycle arrest at the G2/M checkpoint. In addition, *in vivo* findings further confirmed the anti-breast cancer and chemosensitizing effects of ADQ with good safety. Therefore, it is of great value to explore the effects and mechanisms of ADQ in breast cancer.

The development of network pharmacology has shifted our traditional view from the “one drug, one target” model to the “drug–target network” (Hopkins, 2008). Bioinformatics analysis has further optimized the high-throughput screening strategy to validate candidate targets associated with diseases (Eichler, 2012). A target fishing approach has been applied by a number of TCM studies. For example, Wang et al. (2017b) identified tumor-associated macrophages/C-X-C motif chemokine ligand 1 as key modulators of XIAOPI formula in the prevention of breast cancer metastasis based on network pharmacology analysis and cytokine array screening. Chemoinformatics, bioinformatics, and network biology were applied together to predict the active compounds of Tianfoshen oral liquid and to validate its therapeutic targets against colorectal cancer (Wang et al., 2017c). In this study, network analysis revealed a total of 132 active compounds and 297 genes in the ADQ formula, and bioinformatics analysis further identified 22 genes closely correlated with the chemosensitizing activities of ADQ through Venn diagram analysis with breast cancer chemoresponsive genes extracted from GSE41112 and GSE87455. These results suggested that ADQ efficacy was through the synergistic effect of multi-compounds, multi-targets, and multi-pathways. Among these hub molecules, CAV1 was the key target node with the largest “Degree,” indicating that it may be one of the most likely mechanisms affecting the ADQ regulation network. Meanwhile, pathway enrichment analysis demonstrated that CAV1 acted as a key upstream node influencing cell cycle, apoptosis, and p53 signaling. Interestingly, CAV1 has also been considered an important stress-responsive molecule by recent studies and our previous report (Lavie et al., 1998; Yang et al., 1998; Glait et al., 2006; Wang et al., 2015c). Therefore, it was selected for subsequent validation. Interestingly, a lot of work has demonstrated that CAV1 is necessary for p53 activation. For example, Volonte et al. (2009) validated that CAV1 expression is required for the activation of ATM-p53-p21 pathway, and Bartholomew et al. (2009) indicated that CAV1 is a novel binding protein for mouse double minute 2 homolog, thereby preventing p53 proteasome degradation and stabilizing its cellular expression. Our study demonstrated that p53 expression in both MDA-MB-231 and MCF-7 cells was downregulated compared with primary mammary epithelial cells, accompanied by CAV1 reduction (**Supplementary Figure S5**). However, although ADQ administration led to CAV1 inhibition, the expression of p53 and p-p53 (ser15) did not significantly change, indicating that ADQ-induced p21 and apoptosis activation might not be related to p53 (**Supplementary Figure S6**). With regard to the genetic status of p53, MDA-MB-231 was mutated and MCF-7 was wild type. Because the degradation speed of mutated p53 was significantly reduced, p53 expression was highly elevated in MDA-MB-231 cells compared with MCF-7 cells (**Supplementary Figure S5**); however, ADQ had little effect on p53 expression in both breast cancer cells (**Supplementary Figure S6**), indicating that the susceptibility of MDA-MB-231 or MCF-7 cells to ADQ was not related to p53 genetic status. Overall, network pharmacology has provided us with a highly efficient strategy to identify key targets and their complex correlation in the development of TCM formulas.

CAV1 plays dual roles in the progression of breast, lung, cervical, gastric, glioma liver, and prostate cancers (Wang et al., 2017d). During tumor initiation, its loss not only triggers tumor-survival signals including PI3K/Akt and MAPK, but also leads to the inactivation of tumor suppressor genes such as BRCA1 and PTEN (Glait et al., 2006). By contrast, accumulating evidence has suggested that CAV1 overexpression correlates with cancer drug resistance, metastasis, the survival of cancer stem cells, and advanced carcinoma (Wang et al., 2015c). Increased CAV1 expression has also been observed in a series of drug-resistant cancer cells compared with their parental cells such as paclitaxel-resistant A549 cells, vinblastine-resistant SKVLB1 cells, colchicine-resistant HT-29 cells, and adraimycin-resistant MCF-7 cells (Lavie et al., 1998; Yang et al., 1998). Moreover, clinical investigations have revealed that CAV1 expression is positively correlated with chemotherapy response in gastric cancer (Yuan et al., 2013) and non-small lung cancer (Brodie et al., 2014). Therefore targeting CAV1 is a promising strategy for overcoming cancer drug resistance. Interestingly, our study demonstrated that ADQ could inhibit CAV1 to improve breast cancer chemosensitivity. In addition, CAV1-overexpressing MDA-MB-231 cells were more susceptible to ADQ than low CAV1-expressing MCF-7 cells. Consistent with our findings, previous studies have revealed that multiple active compounds in ADQ exerted anti-cancer effects partly by mediating CAV1 expression. For example, it was shown that quercetin reversed tamoxifen resistance in breast cancer cells, and its metabolites likely suppressed CAV1 expression (Wang et al., 2015a; Kamada et al., 2016). Calycosin glycoside regulates nitric oxide/CAV1/matrix metalloproteinase signaling (Fu et al., 2014b). The CAV1-mediated anti-cancer effects of ADQ in this study were possibly due to the complex interaction and synergistic/neutralizing effects among the involved compounds. However, ADQ also exhibited a significant inhibitory effects on MCF-7 cell proliferation, indicating that there may be other molecular targets responsible for the effects of ADQ. Because the phytochemicals in Chinese formula are too complex to analyze, it is unlikely that CAV1 is the only target of ADQ. Our results also demonstrated that CAV1 expression was highly elevated in paclitaxel-resistant breast cancer cells, and ADQ chemosensitized breast cancer cells by inhibiting CAV1, consistent with the oncogenic role of CAV1. This phenomenon might be explained by the property of the stress-related function of CAV1, which plays a key role in protecting cells from hazardous stimuli. During cancer initiation, malignant transformation may be accelerated due to CAV1 loss, which would sensitize normal cells to oncogenic events. In contrast, when cancer progresses and is treated, CAV1 expression may be upregulated to protect cancer cells from escaping death by speeding aerobic glycolysis, increasing stem cell populations, or overexpressing ATP-binding cassette transporters (Wang et al., 2015c).

## Conclusion

The results of our study demonstrated that ADQ improved the cancer chemoresponse, and that CAV1 was at least in part responsible for these chemosensitizing effects. Our work has great implications for the discovery of breast cancer therapeutic targets by integrating bioinformatics and network pharmacology followed by experimental validation. This study not only provides experimental evidence and molecular mechanisms that may facilitate the safe and effective therapeutic use of herbal medicines for breast cancer, and may lead to CAV1-based therapeutic strategies for mammary malignancies. However, preclinical studies are needed to confirm its chemosensitizing effects and active ingredients.

## Author Contributions

NW and ZW conducted the design of the experiments and wrote the manuscript. FZ contributed to the revised manuscript. NW conducted network pharmacology analysis and bioinformatics analysis. BY, XL, and XZ contributed to the drug preparation and quality control of ADQ. SW, YZ, and SL carried out cell culture and molecular biology experiments. ZH, HP, and YL conducted flow cytometry analysis.

## Conflict of Interest Statement

The authors declare that the research was conducted in the absence of any commercial or financial relationships that could be construed as a potential conflict of interest.

## Footnotes

1 http://www.cellresource.cn

2 http://lsp.nwu.edu.cn/tcmsp.php

3 https://academic.oup.com/nar/article/41/D1/D1089/1057998

4 http://bionet.ncpsb.org/batman-tcm/

5 http://www.ncbi.nlm.nih.gov/geo

6 http://www.ncbi.nlm.nih.gov/geo/geo2r/

7 http://david.abcc.ncifcrf.gov/

8 http://www.genome.jp/kegg/kegg2.html

## Supplementary Material

FIGURE S1 The chromatographic fingerprints of ADQ. HPLC chromatograms on 216 nm detection for **(A)** standard compounds, **(B)** ADQ (1: p-Coumaric acid; 2: Calycosin-7-glucoside; 3: Liquiritin; 4: Glycyrrhizic acid; 5: Curcumol).

FIGURE S2 The IC50s of paclitaxel for MDA-MB-231, MDA-MB-231/T, MCF-7, and MCF-7/T cells. The parental/resistant cells of **(A)** MDA-MB-231 and **(B)** MCF-7 were treated with paclitaxel at the indicated concentrations (0–200 ng/mL) for 48 h.

FIGURE S3 The expressions of CAV1 were determined by western blot among MDA-MB-231, MDA-MB-231/T, MCF-7, MCF-7/T, HUMECs, and HUVECs.

FIGURE S4 The expressions of CAV1 using western blotting analysis. **(A)** MCF-7 cells were transfected with the recombinant plasmid of CAV1, and MDA-MB-231 cells were transfected with si*CAV1* for 48 h. The CAV1 levels were then confirmed by western blot analysis; **(B)** The expressions of CAV1 on the indicated parental breast cancer cells and the paired paclitaxel-resistant cells (^∗∗^*P* < 0.01 *v.s.* control, values represented as the mean ± SD, *n* = 3).

FIGURE S5 The expressions of CAV1, p53 and p-p53 (ser15) were determined by western blot among MDA-MB-231, MCF-7, and HUMECs (^∗∗^*P* < 0.01 *v.s.* control, values represented as the mean ± SD, *n* = 3).

FIGURE S6 The expressions of CAV1, p53 and p-p53 (ser15) were determined by western blot with or without ADQ in MDA-MB-231 and MCF-7 (^∗∗^*P* < 0.01 *v.s.* control, values represented as the mean ± SD, *n* = 3).

TABLE S1 The establishment of calibration curves for HPLC analysis.

TABLE S2 The contents of five components in ADQ.

TABLE S3 Information on candidate active compounds from BHSSC, EZ, HQ and GC herbs of ADQ decoration.

## Abbreviations

ADQ: Ai Du Qing
CAV1: caveolin-1
DAD: diode array detection
DAPI: 4′,6-diamidino-2-phenylindole
DAVID: Database for Annotation, Visualization and Integrated Discovery
DEGs: differentially expressed genes
DL: drug-likeness
GEO: Gene Expression Omnibus
GO: Gene Ontology
H&E staining: hematoxylin-eosin staining
HPLC: high-performance liquid chromatography
HUMECs: the primary human mammary epithelial cells
HUVECs: the human umbilical vein endothelial cells
IC50: the half maximal inhibitory concentration
IHC: immunohistochemistry
KEGG: Kyoto Encyclopedia of Genes and Genomes
NCBI: National Center for Biotechnology Information
OB: oral bioavailability
PARP1: Poly (ADP-ribose) polymerase 1
PI: propidium iodide
PPI: protein–protein interaction
TCM: traditional Chinese medicine
TCMID: Traditional Chinese Medicine Integrated Database
TCMSP: Traditional Chinese Medicine Systems Pharmacology
TUNEL: terminal dexynucleotidyl transferase(TdT)-mediated dUTP nick end labeling

## References

[B1] Bartholomew J. N. Volonte D. Galbiati F. (2009). Caveolin-1 regulates the antagonistic pleiotropic properties of cellular senescence through a novel Mdm2/p53-mediated pathway. *Cancer Res.* 69 2878–2886. 10.1158/0008-5472.CAN-08-2857 19318577 PMC2692066

[B2] Brodie S. A. Lombardo C. Li G. Kowalski J. Gandhi K. You S. (2014). Aberrant promoter methylation of caveolin-1 is associated with favorable response to taxane-platinum combination chemotherapy in advanced NSCLC. *PLoS One* 9:e107124. 10.1371/journal.pone.0107124 25222296 PMC4164573

[B3] DeSantis C. E. Ma J. Goding Sauer A. Newman L. A. Jemal A. (2017). Breast cancer statistics, 2017, racial disparity in mortality by state. *CA Cancer J. Clin.* 67 439–448. 10.3322/caac.21412 28972651

[B4] Eichler G. S. (2012). Bioinformatics/biostatistics: microarray analysis. *Methods Mol. Biol.* 823 347-358. 10.1007/978-1-60327-216-2_22 22081356

[B5] Fan T. P. Yeh J. C. Leung K. W. Yue P. Y. Wong R. N. (2006). Angiogenesis: from plants to blood vessels. *Trends Pharmacol. Sci.* 27 297–309. 10.1016/j.tips.2006.04.006 16697473

[B6] Feng J. Jin Y. Peng J. Wei L. Cai Q. Yan Z. (2017). *Hedyotis Diffusa* Willd extract suppresses colorectal cancer growth through multiple cellular pathways. *Oncol. Lett.* 14 8197–8205. 10.3892/ol.2017.7244 29344262 PMC5755052

[B7] Fu J. Wang Z. Huang L. Zheng S. Wang D. Chen S. (2014a). Review of the botanical characteristics, phytochemistry, and pharmacology of *Astragalus membranaceus* (Huangqi). *Phytother. Res.* 28 1275–1283. 10.1002/ptr.5188 25087616

[B8] Fu S. Gu Y. Jiang J. Q. Chen X. Xu M. Shen J. (2014b). Calycosin-7-O-beta-D-glucoside regulates nitric oxide /caveolin-1/matrix metalloproteinases pathway and protects blood-brain barrier integrity in experimental cerebral ischemia-reperfusion injury. *J. Ethnopharmacol.* 155 692–701. 10.1016/j.jep.2014.06.015 24930357

[B9] Gao X. F. Li Q. L. Li H. L. Zhang H. Y. Su J. Y. Wang B. (2014). Extracts from *Curcuma zedoaria* inhibit proliferation of human breast cancer cell MDA-MB-231 in vitro. *Evid. Based Complement. Alternat. Med.* 2014:730678. 10.1155/2014/730678 24883070 PMC4026840

[B10] Glait C. Ravid D. Lee S. W. Liscovitch M. Werner H. (2006). Caveolin-1 controls BRCA1 gene expression and cellular localization in human breast cancer cells. *FEBS Lett.* 580 5268–5274. 10.1016/j.febslet.2006.08.071 16979166

[B11] Gupta S. C. Prasad S. Sethumadhavan D. R. Nair M. S. Mo Y. Y. Aggarwal B. B. (2013). Nimbolide, a limonoid triterpene, inhibits growth of human colorectal cancer xenografts by suppressing the proinflammatory microenvironment. *Clin. Cancer Res.* 19 4465–4476. 10.1158/1078-0432.CCR-13-0080 23766363 PMC4220790

[B12] Hopkins A. L. (2008). Network pharmacology: the next paradigm in drug discovery. *Nat. Chem. Biol.* 4 682–690. 10.1038/nchembio.118 18936753

[B13] Janczar S. Nautiyal J. Xiao Y. Curry E. Sun M. Zanini E. (2017). WWOX sensitises ovarian cancer cells to paclitaxel via modulation of the ER stress response. *Cell Death Dis.* 8:e2955. 10.1038/cddis.2017.346 28749468 PMC5550887

[B14] Johnstone R. W. Ruefli A. A. Lowe S. W. (2002). Apoptosis: a link between cancer genetics and chemotherapy. *Cell* 108 153–164. 11832206 10.1016/s0092-8674(02)00625-6

[B15] Kamada C. Mukai R. Kondo A. Sato S. Terao J. (2016). Effect of quercetin and its metabolite on caveolin-1 expression induced by oxidized LDL and lysophosphatidylcholine in endothelial cells. *J. Clin. Biochem. Nutr.* 58 193–201. 10.3164/jcbn.16-2 27257344 PMC4865600

[B16] Lam W. Jiang Z. Guan F. Huang X. Hu R. Wang J. (2015). PHY906(KD018), an adjuvant based on a 1800-year-old Chinese medicine, enhanced the anti-tumor activity of sorafenib by changing the tumor microenvironment. *Sci. Rep.* 5:9384. 10.1038/srep09384 25819872 PMC4377583

[B17] Lavie Y. Fiucci G. Liscovitch M. (1998). Up-regulation of caveolae and caveolar constituents in multidrug-resistant cancer cells. *J. Biol. Chem.* 273 32380–32383. 9829965 10.1074/jbc.273.49.32380

[B18] Lin E. Y. Jones J. G. Li P. Zhu L. Whitney K. D. Muller W. J. (2003). Progression to malignancy in the polyoma middle T oncoprotein mouse breast cancer model provides a reliable model for human diseases. *Am. J. Pathol.* 163 2113–2126. 10.1016/s0002-9440(10)63568-7 14578209 PMC1892434

[B19] Li P. Fu Y. Ru J. Huang C. Du J. Zheng C. (2014). Insights from systems pharmacology into cardiovascular drug discovery and therapy. *BMC Syst. Biol.* 8:141. 10.1186/s12918-014-0141-z 25539592 PMC4297424

[B20] Li Q. Q. Wang G. Huang F. Banda M. Reed E. (2010). Antineoplastic effect of beta-elemene on prostate cancer cells and other types of solid tumour cells. *J. Pharm. Pharmacol.* 62 1018–1027. 10.1111/j.2042-7158.2010.01135.x 20663036

[B21] Liu H. Wang J. Zhou W. Wang Y. Yang L. (2013). Systems approaches and polypharmacology for drug discovery from herbal medicines: an example using licorice. *J. Ethnopharmacol.* 146 773–793. 10.1016/j.jep.2013.02.004 23415946

[B22] Li X. Wang G. Zhao J. Ding H. Cunningham C. Chen F. (2005). Antiproliferative effect of beta-elemene in chemoresistant ovarian carcinoma cells is mediated through arrest of the cell cycle at the G2-M phase. *Cell. Mol. Life Sci.* 62 894–904. 10.1007/s00018-005-5027-1 15868412 PMC11924550

[B23] Lu Z. J. (1985). The yellow emperor’s internal classic, an ancient medical canon of traditional Chinese medicine. *J. Tradit. Chin. Med.* 5 153–154. 3903358

[B24] Mello S. S. Attardi L. D. (2017). Deciphering p53 signaling in tumor suppression. *Curr. Opin. Cell Biol.* 51 65–72. 10.1016/j.ceb.2017.11.005 29195118 PMC5949255

[B25] Qi F. Zhao L. Zhou A. Zhang B. Li A. Wang Z. (2015). The advantages of using traditional Chinese medicine as an adjunctive therapy in the whole course of cancer treatment instead of only terminal stage of cancer. *Biosci. Trends* 9 16–34. 10.5582/bst.2015.01019 25787906

[B26] Ru J. Li P. Wang J. Zhou W. Li B. Huang C. (2014). TCMSP: a database of systems pharmacology for drug discovery from herbal medicines. *J. Cheminform.* 6:13. 10.1186/1758-2946-6-13 24735618 PMC4001360

[B27] Sancar A. Lindsey-Boltz L. A. Unsal-Kacmaz K. Linn S. (2004). Molecular mechanisms of mammalian DNA repair and the DNA damage checkpoints. *Annu. Rev. Biochem.* 73 39–85. 10.1146/annurev.biochem.73.011303.073723 15189136

[B28] Shi Y. Tan S. H. Ng S. Zhou J. Yang N. D. Koo G. B. (2015). Critical role of CAV1/caveolin-1 in cell stress responses in human breast cancer cells via modulation of lysosomal function and autophagy. *Autophagy* 11 769–784. 10.1080/15548627.2015.1034411 25945613 PMC4509445

[B29] Song L. M. Wu J. R. Jiang D. (2015). Analysis of the medication rules of national TCM masters for the treatment of phymatosis based on data mining. *Chin. J. Inf. TCM* 22 50–53.

[B30] Stage T. B. Bergmann T. K. Kroetz D. L. (2018). Clinical pharmacokinetics of paclitaxel monotherapy: an updated literature review. *Clin. Pharmacokinet.* 57 7–19. 10.1007/s40262-017-0563-z 28612269 PMC8572663

[B31] Volonte D. Kahkonen B. Shapiro S. Di Y. Galbiati F. (2009). Caveolin-1 expression is required for the development of pulmonary emphysema through activation of the ATM-p53-p21 pathway. *J. Biol. Chem.* 284 5462–5466. 10.1074/jbc.C800225200 19103597 PMC2645811

[B32] Wang C. Schwab L. P. Fan M. Seagroves T. N. Buolamwini J. K. (2013). Chemoprevention activity of dipyridamole in the MMTV-PyMT transgenic mouse model of breast cancer. *Cancer Prev. Res.* 6 437–447. 10.1158/1940-6207.capr-12-0345 23447563 PMC3829204

[B33] Wang H. Tao L. Qi K. Zhang H. Feng D. Wei W. (2015a). Quercetin reverses tamoxifen resistance in breast cancer cells. *J. BUON* 20 707–713. 26214621

[B34] Wang N. Wang Q. Tang H. Zhang F. Zheng Y. Wang S. (2017a). Direct inhibition of ACTN4 by ellagic acid limits breast cancer metastasis via regulation of beta-catenin stabilization in cancer stem cells. *J. Exp. Clin. Cancer Res.* 36:172. 10.1186/s13046-017-0635-9 29197410 PMC5712102

[B35] Wang N. Wang Z. Peng C. You J. Shen J. Han S. (2014). Dietary compound isoliquiritigenin targets GRP78 to chemosensitize breast cancer stem cells via beta-catenin/ABCG2 signaling. *Carcinogenesis* 35 2544–2554. 10.1093/carcin/bgu187 25194164

[B36] Wang N. Wang Z. Wang Y. Xie X. Shen J. Peng C. (2015b). Dietary compound isoliquiritigenin prevents mammary carcinogenesis by inhibiting breast cancer stem cells through WIF1 demethylation. *Oncotarget* 6 9854–9876. 10.18632/oncotarget.3396 25918249 PMC4496402

[B37] Wang N. Zheng Y. Gu J. Cai Y. Wang S. Zhang F. (2017b). Network-pharmacology-based validation of TAMS/CXCL-1 as key mediator of XIAOPI formula preventing breast cancer development and metastasis. *Sci. Rep.* 7:14513. 10.1038/s41598-017-15030-3 29109519 PMC5674025

[B38] Wang S. Wang H. Lu Y. (2017c). Tianfoshen oral liquid: a CFDA approved clinical traditional Chinese medicine, normalizes major cellular pathways disordered during colorectal carcinogenesis. *Oncotarget* 8 14549–14569. 10.18632/oncotarget.14675 28099904 PMC5362425

[B39] Wang S. Wang N. Zheng Y. Zhang J. Zhang F. Wang Z. (2017d). Caveolin-1: an oxidative stress-related target for cancer prevention. *Oxid. Med. Cell. Longev.* 2017:7454031. 10.1155/2017/7454031 28546853 PMC5436035

[B40] Wang Z. Wang N. Liu P. Peng F. Tang H. Chen Q. (2015c). Caveolin-1, a stress-related oncotarget, in drug resistance. *Oncotarget* 6 37135–37150. 10.18632/oncotarget.5789 26431273 PMC4741920

[B41] Xu T. Pi Z. Liu S. Song F. Liu Z. (2017). Chemical profiling combined with “omics” technologies (CP-omics): a strategy to understand the compatibility mechanisms and simplify herb formulas in traditional chinese medicines. *Phytochem. Anal.* 28 381–391. 10.1002/pca.2685 28387961

[B42] Yang C. P. Galbiati F. Volonte D. Horwitz S. B. Lisanti M. P. (1998). Upregulation of caveolin-1 and caveolae organelles in taxol-resistant A549 cells. *FEBS Lett.* 439 368–372. 9845355 10.1016/s0014-5793(98)01354-4

[B43] Yuan G. Regel I. Lian F. Friedrich T. Hitkova I. Hofheinz R. D. (2013). WNT6 is a novel target gene of caveolin-1 promoting chemoresistance to epirubicin in human gastric cancer cells. *Oncogene* 32 375–387. 10.1038/onc.2012.40 22370641

[B44] Zhang J. Guo H. Qian G. Ge S. Ji H. Hu X. (2010). MiR-145, a new regulator of the DNA fragmentation factor-45 (DFF45)-mediated apoptotic network. *Mol. Cancer* 9:211. 10.1186/1476-4598-9-211 20687965 PMC2924312

