# Supplementary figures and images for: Network Pharmacology-Based Validation of Caveolin-1 as a Key Mediator of Ai Du Qing Inhibition of Drug Resistance in Breast Cancer
Source: Front Pharmacol. 2018 Oct 2;9:1106. doi: 10.3389/fphar.2018.01106 (PMC6176282; doi:10.3389/fphar.2018.01106)

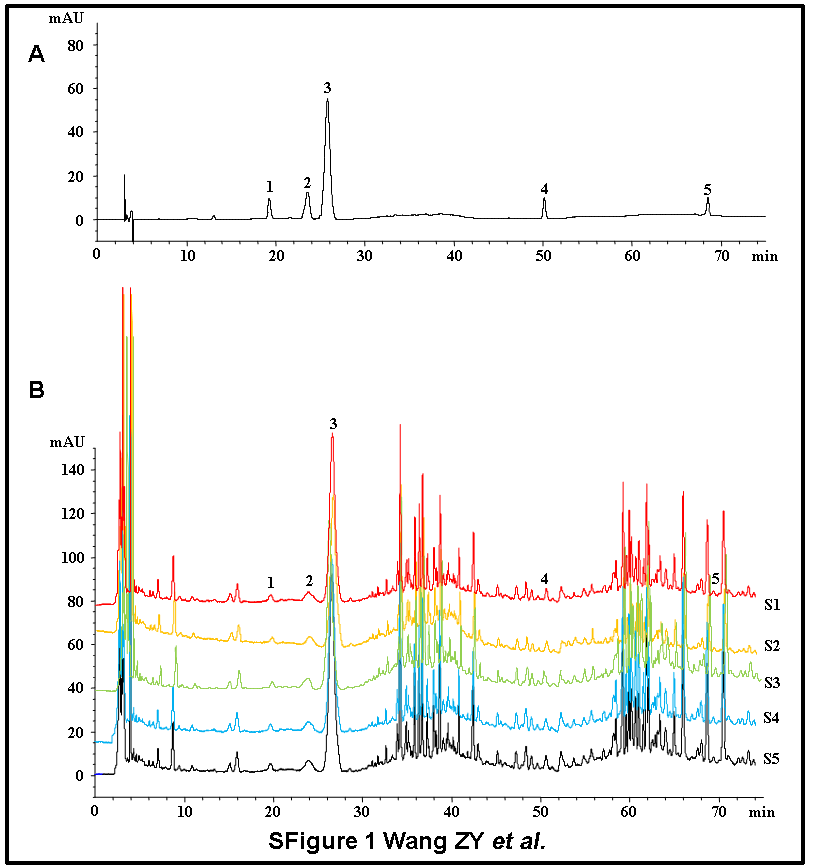

Supplement: FIGURE S1 — The chromatographic fingerprints of ADQ. HPLC chromatograms on 216 nm detection for (A) standard compounds, (B) ADQ (1: p-Coumaric acid; 2: Calycosin-7-glucoside; 3: Liquiritin; 4: Glycyrrhizic acid; 5: Curcumol). [file Image_1.TIF]

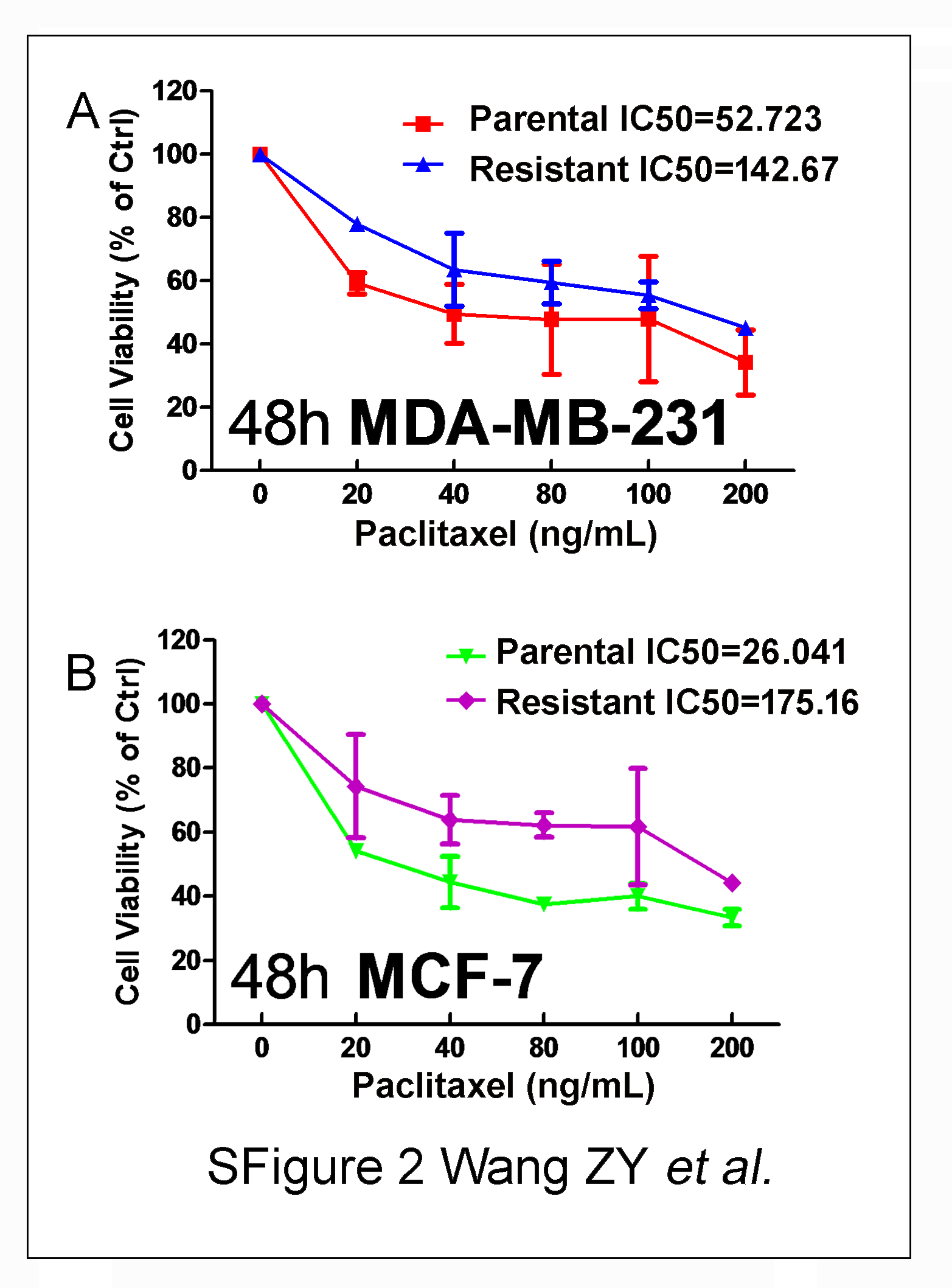

Supplement: FIGURE S2 — The IC50s of paclitaxel for MDA-MB-231, MDA-MB-231/T, MCF-7, and MCF-7/T cells. The parental/resistant cells of (A) MDA-MB-231 and (B) MCF-7 were treated with paclitaxel at the indicated concentrations (0–200 ng/mL) for 48 h. [file Image_2.TIF]

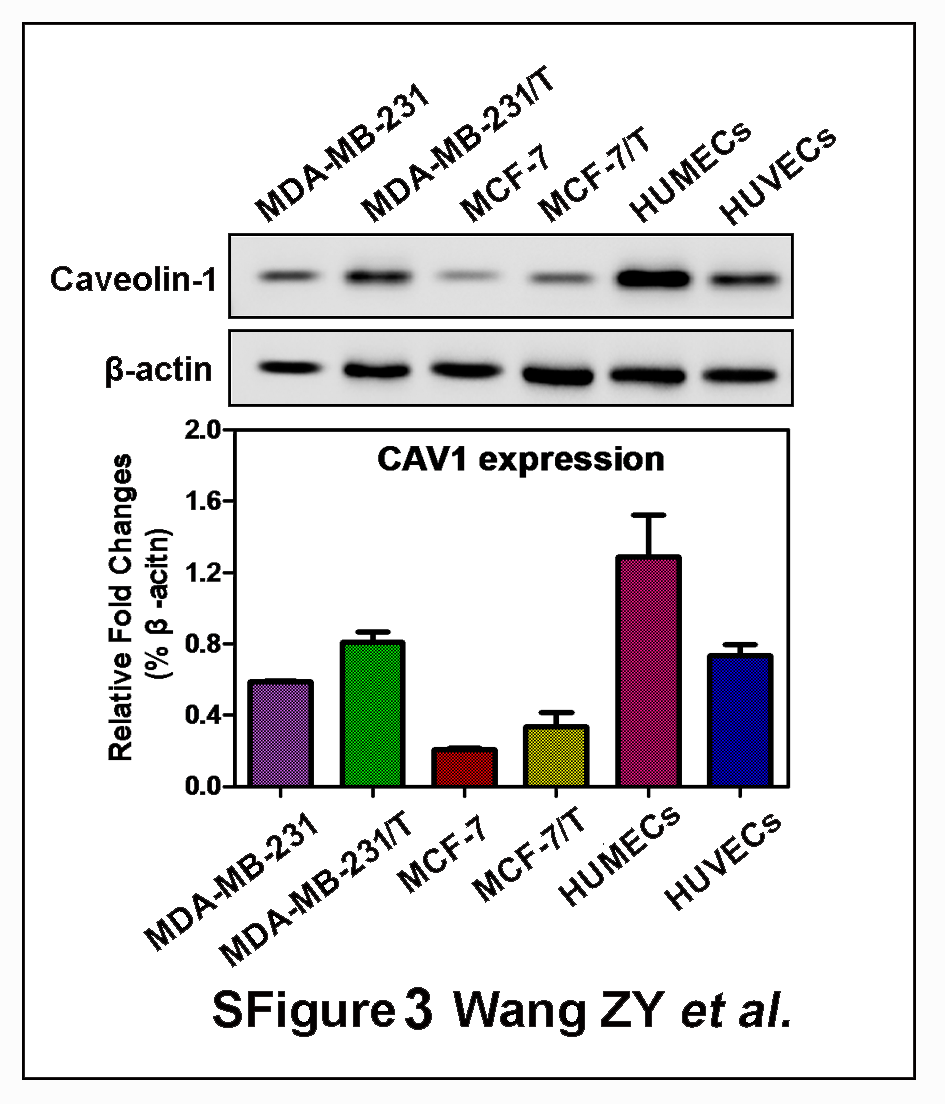

Supplement: FIGURE S3 — The expressions of CAV1 were determined by western blot among MDA-MB-231, MDA-MB-231/T, MCF-7, MCF-7/T, HUMECs, and HUVECs. [file Image_3.TIF]

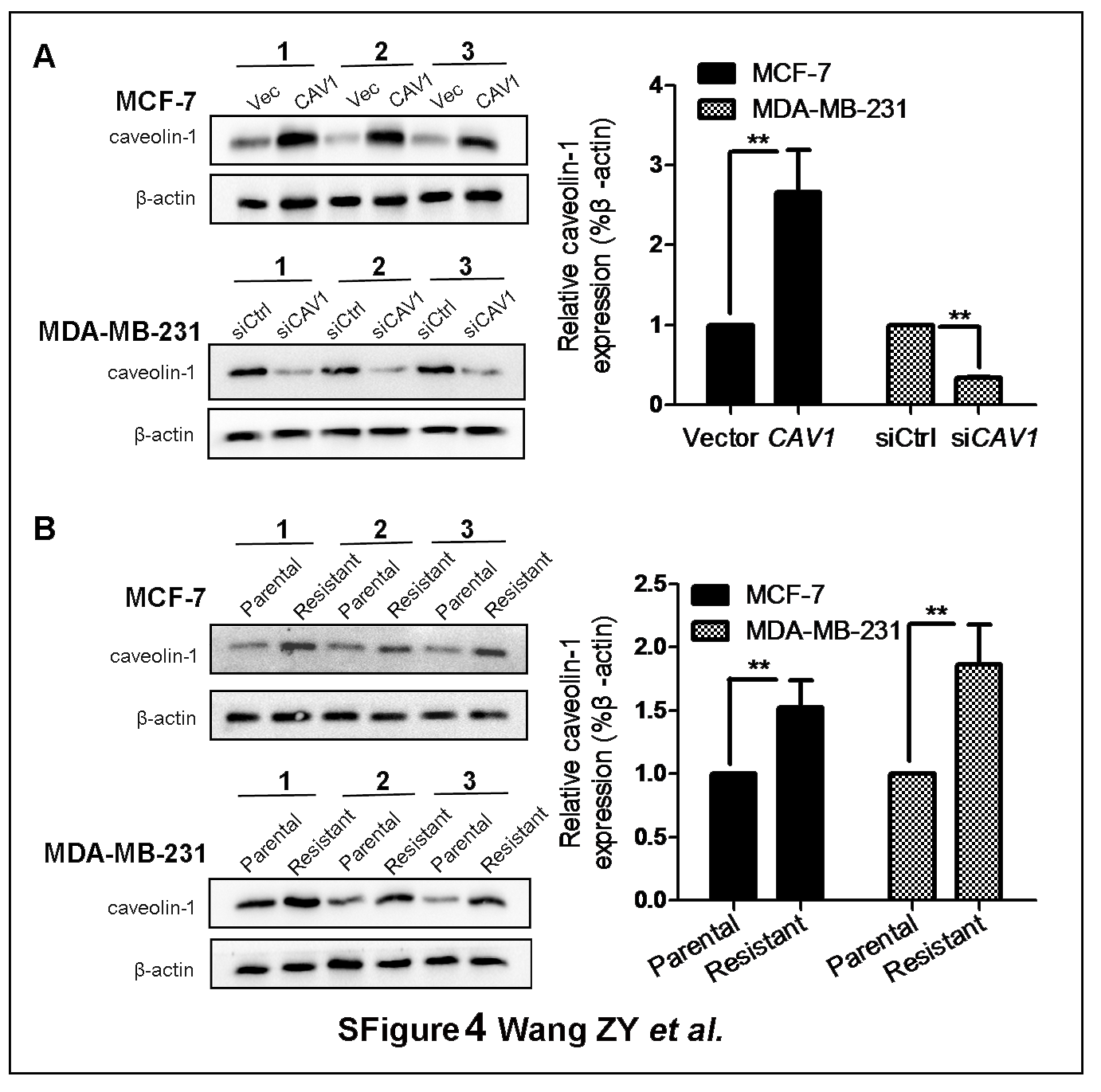

Supplement: FIGURE S4 — The expressions of CAV1 using western blotting analysis. (A) MCF-7 cells were transfected with the recombinant plasmid of CAV1, and MDA-MB-231 cells were transfected with siCAV1 for 48 h. The CAV1 levels were then confirmed by western blot analysis; (B) The expressions of CAV1 on the indicated parental breast cancer cells and the paired paclitaxel-resistant cells (∗∗P < 0.01 v.s. control, values represented as the mean ± SD, n = 3). [file Image_4.TIF]

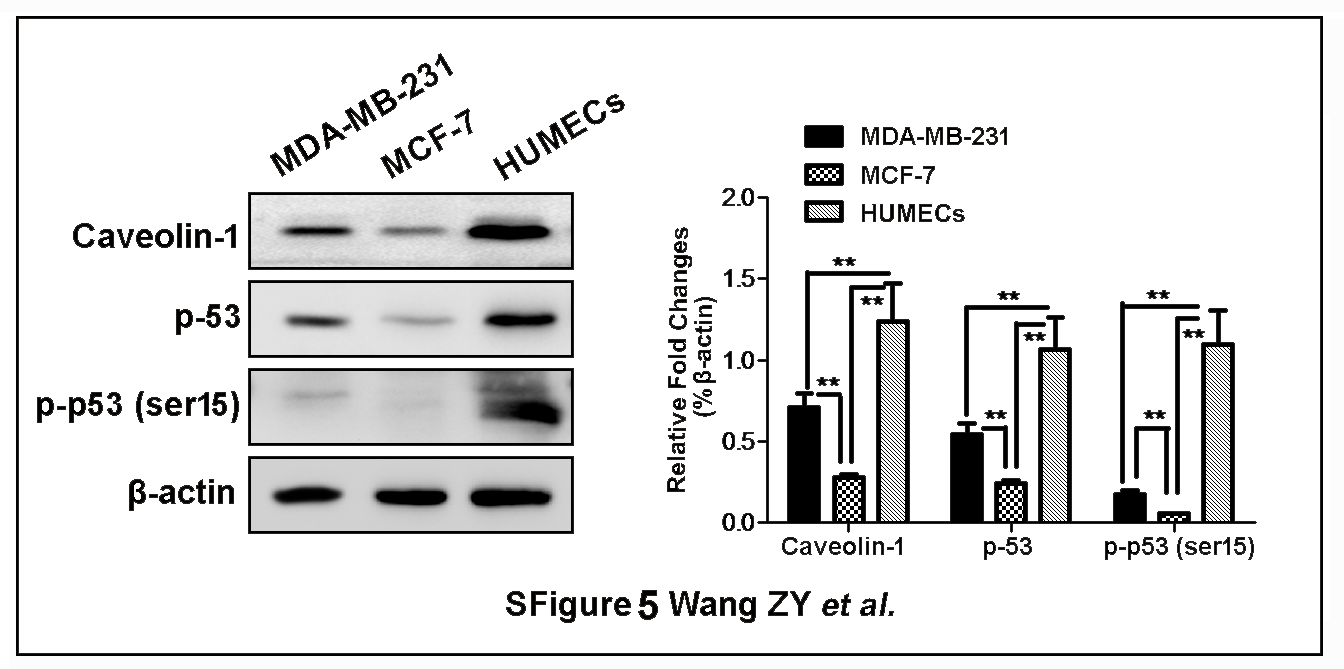

Supplement: FIGURE S5 — The expressions of CAV1, p53 and p-p53 (ser15) were determined by western blot among MDA-MB-231, MCF-7, and HUMECs (∗∗P < 0.01 v.s. control, values represented as the mean ± SD, n = 3). [file Image_5.TIF]

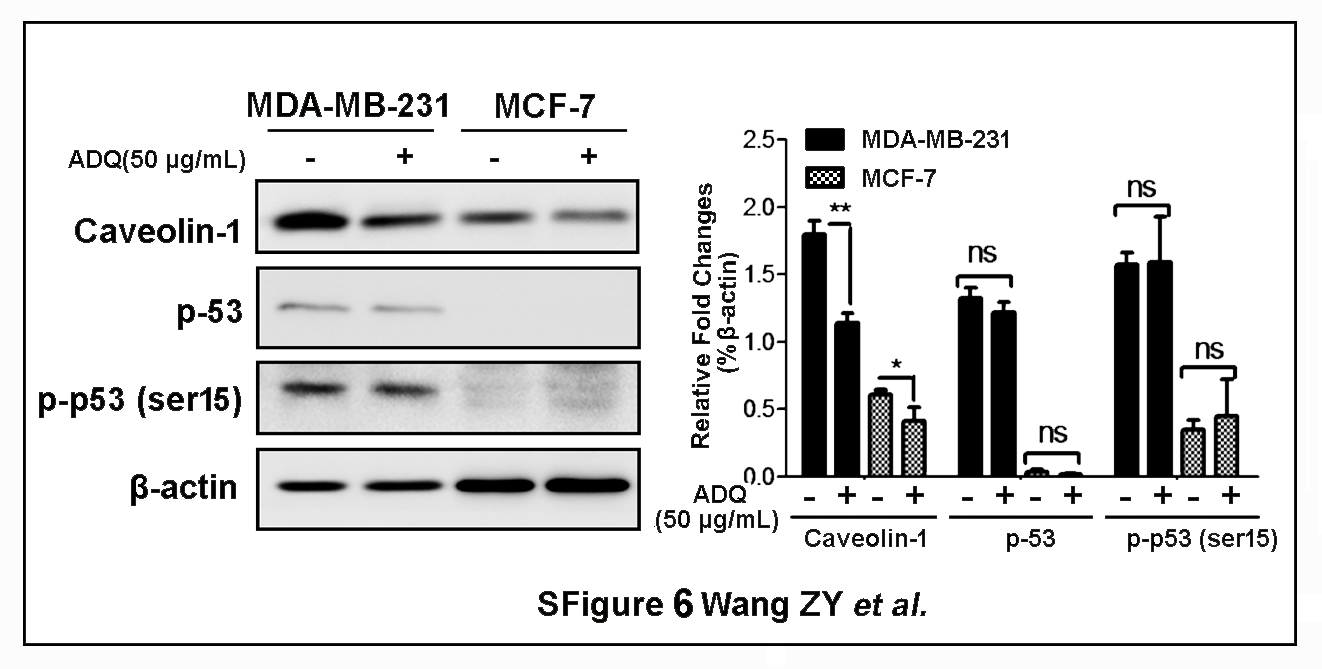

Supplement: FIGURE S6 — The expressions of CAV1, p53 and p-p53 (ser15) were determined by western blot with or without ADQ in MDA-MB-231 and MCF-7 (∗∗P < 0.01 v.s. control, values represented as the mean ± SD, n = 3). [file Image_6.TIF]
