# Supplementary material for: Network Pharmacology-Based Validation of Caveolin-1 as a Key Mediator of Ai Du Qing Inhibition of Drug Resistance in Breast Cancer
Source: Front Pharmacol. 2018 Oct 2;9:1106. doi: 10.3389/fphar.2018.01106 (PMC6176282; doi:10.3389/fphar.2018.01106)
Supplement: TABLE S1 — The establishment of calibration curves for HPLC analysis. [file Table_1.DOC]

**Supplementary Tables:**

**Supplementary Table 1:** The establishment of calibration curves for HPLC analysis.

| **Analyte** | **Calibration curve** | ***R^2^*** | **Linear range(μg/mL)** |
| --- | --- | --- | --- |
| *p*-Coumaric acid | *Y* = 69.996*X* - 5.5249 | 0.9999 | 1.05~21.0 |
| Calycosin-7-glucoside | *Y* = 16.703*X* + 8.8779 | 0.9997 | 4.0~40.0 |
| Liquiritin | *Y* = 17.059*X* + 15.766 | 0.9995 | 10.0~200 |
| Glycyrrhizic acid | *Y* = 6.961*X*- 5.3055 | 0.9997 | 10.5~210 |
| Curcumol | *Y* = 4.5638*X* + 0.0903 | 0.9998 | 1.1~22.0 |

**Supplementary Table 2:** The Contents of Five Components in ADQ.

|  | **Contents (mg/g)** | | | | |
| --- | --- | --- | --- | --- | --- |
|  | **p-Coumaric acid** | **Calycosin-7-glucoside** | **Liquiritin** | **Glycyrrhizic acid** | **Curcumol** |
| 1 | 0.4323 | 1.3934 | 22.1439 | 14.1081 | 1.7293 |
| 2 | 0.4417 | 1.7323 | 22.7694 | 14.1838 | 1.1414 |
| 3 | 0.4410 | 1.9672 | 22.5299 | 11.0368 | 5.2054 |
| 4 | 0.4065 | 1.4825 | 21.0420 | 10.1345 | 3.1234 |
| 5 | 0.4331 | 1.7981 | 21.2149 | 11.4325 | 2.1653 |

**Supplementary Table 3:** Information on candidate active compounds from BHSSC, EZ, HQ and GC herbs of ADQ decoration.

| **Chinese Name** | **Latin name** | **Number of Screening ingredients** | **Major ingredients** | **Number of Targets** |
| --- | --- | --- | --- | --- |
|  |  |  |  |  |
|  |  |  |  |  |
| 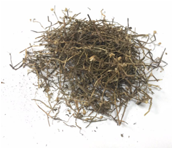白花蛇舌草 | | *Hedyotis diffusa* | | --- | | | 12 | deacetyl asperuloside acid_qt | 225 |
|  |  |  | quercetin |  |
|  |  |  | Poriferasterol |  |
|  |  |  | Stigmasterol |  |
|  |  |  | 2-methoxy-3-methyl-9,10-anthraquinone |  |
|  |  |  | beta-sitosterol |  |
|  |  |  | deacetylasperulosidic acid _qt |  |
|  |  |  | scandoside_qt |  |
|  |  |  | Geniposidic acid |  |
|  |  |  | rutin |  |
|  |  |  | ursolic acid |  |
|  |  |  | p-coumaric acid |  |
| 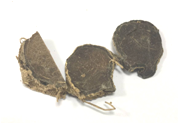莪 术 | | *Curcuma zedoaria (Christm.) Rosc* | | --- | | | 14 | ()-Aromadendrene | 41 |
|  |  |  | hederagenin |  |
|  |  |  | (-)-Epoxycaryophyllene |  |
|  |  |  | isocurcumenol |  |
|  |  |  | (1R,10R)-epoxy-1,10-dihydrocurdione |  |
|  |  |  | (3S,3aR,8aR)-3,8a-dihydroxy-5-isopropylidene-3,8-dimethyl-1,2,3a,4-tetrahydroazulen-6-one |  |
|  |  |  | (5R,6R)-5-isopropenyl-3,6-dimethyl-6-vinyl-5,7-dihydrobenzofuran-4-one |  |
|  |  |  | BRN 3094585 |  |
|  |  |  | curcumol |  |
|  |  |  | Gweicurculactone |  |
|  |  |  | (1S,10S),(4S,5S)-germacrone-1(10),4-diepoxide |  |
|  |  |  | Hepanal |  |
|  |  |  | 58870_FLUKA |  |
|  |  |  | calarene |  |
| 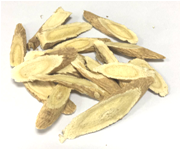黄 芪 | | *Astragalus membranaceus (Fisch.) Bunge.* | | --- | | | 21 | EIC | 222 |
|  |  |  | Mairin |  |
|  |  |  | Jaranol |  |
|  |  |  | hederagenin |  |
|  |  |  | (3S,8S,9S,10R,13R,14S,17R)-10,13-dimethyl-17-[(2R,5S)-5-propan-2-yloctan-2-yl]-2,3,4,7,8,9,11,12,14,15,16,17-dodecahydro-1H-cyclopenta[a]phenanthren-3-ol |  |
|  |  |  | isorhamnetin |  |
|  |  |  | 3,9-di-O-methylnissolin |  |
|  |  |  | 7-O-methylisomucronulatol |  |
|  |  |  | 9,10-dimethoxypterocarpan-3-O-β-D-glucoside |  |
|  |  |  | (6aR,11aR)-9,10-dimethoxy-6a,11a-dihydro-6H-benzofurano[3,2-c]chromen-3-ol |  |
|  |  |  | 13-hydroxy-9,11-octadecadienoic acid |  |
|  |  |  | Bifendate |  |
|  |  |  | formononetin |  |
|  |  |  | Calycosin |  |
|  |  |  | kaempferol |  |
|  |  |  | linolenic acid |  |
|  |  |  | FA |  |
|  |  |  | (Z)-1-(2,4-dihydroxyphenyl)-3-(4-hydroxyphenyl)prop-2-en-1-one |  |
|  |  |  | isomucronulatol-7,2'-di-O-glucosiole |  |
|  |  |  | 1,7-Dihydroxy-3,9-dimethoxy pterocarpene |  |
|  |  |  | quercetin |  |
| 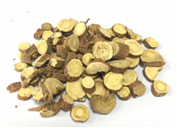甘 草 | | *Glycyrrhiza uralensis Fisch.* | | --- | | | 94 | Inermine | 241 |
|  |  |  | isoliquiritigenin |  |
|  |  |  | DFV |  |
|  |  |  | Mairin |  |
|  |  |  | Glycyrol |  |
|  |  |  | Jaranol |  |
|  |  |  | Medicarpin |  |
|  |  |  | Pinocembrin |  |
|  |  |  | isorhamnetin |  |
|  |  |  | sitosterol |  |
|  |  |  | Lupiwighteone |  |
|  |  |  | 7-Methoxy-2-methyl isoflavone |  |
|  |  |  | formononetin |  |
|  |  |  | Calycosin |  |
|  |  |  | kaempferol |  |
|  |  |  | naringenin |  |
|  |  |  | (2S)-2-[4-hydroxy-3-(3-methylbut-2-enyl)phenyl]-8,8-dimethyl-2,3-dihydropyrano[2,3-f]chromen-4-one |  |
|  |  |  | euchrenone |  |
|  |  |  | glyasperin B |  |
|  |  |  | glyasperin F |  |
|  |  |  | Glyasperin C |  |
|  |  |  | Isotrifoliol |  |
|  |  |  | (E)-1-(2,4-dihydroxyphenyl)-3-(2,2-dimethylchromen-6-yl)prop-2-en-1-one |  |
|  |  |  | kanzonols W |  |
|  |  |  | (2S)-6-(2,4-dihydroxyphenyl)-2-(2-hydroxypropan-2-yl)-4-methoxy-2,3-dihydrofuro[3,2-g]chromen-7-one |  |
|  |  |  | Semilicoisoflavone B |  |
|  |  |  | Glepidotin A |  |
|  |  |  | Glepidotin B |  |
|  |  |  | Phaseolinisoflavan |  |
|  |  |  | Glypallichalcone |  |
|  |  |  | echinatin |  |
|  |  |  | 8-(6-hydroxy-2-benzofuranyl)-2,2-dimethyl-5-chromenol |  |
|  |  |  | Licochalcone B |  |
|  |  |  | licochalcone G |  |
|  |  |  | 3-(2,4-dihydroxyphenyl)-8-(1,1-dimethylprop-2-enyl)-7-hydroxy-5-methoxy-coumarin |  |
|  |  |  | Licoricone |  |
|  |  |  | Gancaonin A |  |
|  |  |  | Gancaonin B |  |
|  |  |  | 3-(3,4-dihydroxyphenyl)-5,7-dihydroxy-8-(3-methylbut-2-enyl)chromone |  |
|  |  |  | 5,7-dihydroxy-3-(4-methoxyphenyl)-8-(3-methylbut-2-enyl)chromone |  |
|  |  |  | 2-(3,4-dihydroxyphenyl)-5,7-dihydroxy-6-(3-methylbut-2-enyl)chromone |  |
|  |  |  | Glycyrin |  |
|  |  |  | Licocoumarone |  |
|  |  |  | Licoisoflavone |  |
|  |  |  | Licoisoflavone B |  |
|  |  |  | licoisoflavanone |  |
|  |  |  | shinpterocarpin |  |
|  |  |  | (E)-3-[3,4-dihydroxy-5-(3-methylbut-2-enyl)phenyl]-1-(2,4-dihydroxyphenyl)prop-2-en-1-one |  |
|  |  |  | liquiritin |  |
|  |  |  | licopyranocoumarin |  |
|  |  |  | Glyzaglabrin |  |
|  |  |  | Glabridin |  |
|  |  |  | Glabranin |  |
|  |  |  | Glabrene |  |
|  |  |  | Glabrone |  |
|  |  |  | 1,3-dihydroxy-9-methoxy-6-benzofurano[3,2-c]chromenone |  |
|  |  |  | 1,3-dihydroxy-8,9-dimethoxy-6-benzofurano[3,2-c]chromenone |  |
|  |  |  | Eurycarpin A |  |
|  |  |  | (-)-Medicocarpin |  |
|  |  |  | Sigmoidin-B |  |
|  |  |  | (2R)-7-hydroxy-2-(4-hydroxyphenyl)chroman-4-one |  |
|  |  |  | (2S)-7-hydroxy-2-(4-hydroxyphenyl)-8-(3-methylbut-2-enyl)chroman-4-one |  |
|  |  |  | Isoglycyrol |  |
|  |  |  | Isolicoflavonol |  |
|  |  |  | HMO |  |
|  |  |  | 1-Methoxyphaseollidin |  |
|  |  |  | Quercetin der. |  |
|  |  |  | (Z)-1-(2,4-dihydroxyphenyl)-3-phenylprop-2-en-1-one |  |
|  |  |  | 3'-Hydroxy-4'-O-Methylglabridin |  |
|  |  |  | licochalcone a |  |
|  |  |  | 3'-Methoxyglabridin |  |
|  |  |  | 2-[(3R)-8,8-dimethyl-3,4-dihydro-2H-pyrano[6,5-f]chromen-3-yl]-5-methoxyphenol |  |
|  |  |  | Inflacoumarin A |  |
|  |  |  | icos-5-enoic acid |  |
|  |  |  | Kanzonol F |  |
|  |  |  | 6-prenylated eriodictyol |  |
|  |  |  | 7,2',4'-trihydroxy－5-methoxy-3－arylcoumarin |  |
|  |  |  | 7-Acetoxy-2-methylisoflavone |  |
|  |  |  | 8-prenylated eriodictyol |  |
|  |  |  | gadelaidic acid |  |
|  |  |  | Vestitol |  |
|  |  |  | Gancaonin G |  |
|  |  |  | Gancaonin H |  |
|  |  |  | Licoagrocarpin |  |
|  |  |  | Glyasperins M |  |
|  |  |  | Glycyrrhiza flavonol A |  |
|  |  |  | Licoagroisoflavone |  |
|  |  |  | Odoratin |  |
|  |  |  | Phaseol |  |
|  |  |  | Xambioona |  |
|  |  |  | dehydroglyasperins C |  |
|  |  |  | DIBP |  |
|  |  |  | DBP |  |
|  |  |  | quercetin |  |
| TCMSP screening criteria: OB: ≥30%; DL ≥0.1 | | | | |
|  |  |  |  |  |
